# LncRNA LINC00969 promotes acquired gefitinib resistance by epigenetically suppressing of NLRP3 at transcriptional and posttranscriptional levels to inhibit pyroptosis in lung cancer

**DOI:** 10.1038/s41419-023-05840-x

**Published:** 2023-05-08

**Authors:** Jiali Dai, Tianyu Qu, Dandan Yin, Yanan Cui, Chen Zhang, Erbao Zhang, Renhua Guo

**Affiliations:** 1 Department of Oncology, First Affiliated Hospital of Nanjing Medical University, Nanjing, Jiangsu 210029 P. R. China; 2 Department of Respiratory Medicine, Zhongda Hospital of Southeast University, Nanjing, Jiangsu 210009 P. R. China; 3 Clinical Research Center, The Second Hospital of Nanjing, Nanjing University of Chinese Medicine, Nanjing, Jiangsu 210003 P. R. China; 4 Department of Epidemiology, Center for Global Health, School of Public Health, Nanjing Medical University, Nanjing, 211166 P. R. China; 5 Jiangsu Key Lab of Cancer Biomarkers, Prevention and Treatment, Collaborative Innovation Center for Cancer Personalized Medicine, Nanjing Medical University, Nanjing, 211166 P. R. China

**Keywords:** Non-small-cell lung cancer, Long non-coding RNAs, Post-translational modifications, RNA modification

## Abstract

Epidermal growth factor receptor-tyrosine kinase inhibitor (EGFR-TKI) treatment prolongs the survival of lung cancer patients harbouring activating EGFR mutations. However, resistance to EGFR-TKIs is inevitable after long-term treatment. Molecular mechanistic research is of great importance in combatting resistance. A comprehensive investigation of the molecular mechanisms underlying resistance has important implications for overcoming resistance. An accumulating body of evidence shows that lncRNAs can contribute to tumorigenesis and treatment resistance. By bioinformatics analysis, we found that LINC00969 expression was elevated in lung cancer cells with acquired gefitinib resistance. LINC00969 regulated resistance to gefitinib in vitro and in vivo. Mechanistically, gain of H3K4me1 and H3K27Ac led to the activation of LINC00969 expression. LINC00969 interacts with EZH2 and METTL3, transcriptionally regulates the level of H3K27me3 in the NLRP3 promoter region, and posttranscriptionally modifies the m6A level of NLRP3 in an m6A-YTHDF2-dependent manner, thus epigenetically repressing NLRP3 expression to suppress the activation of the NLRP3/caspase-1/GSDMD-related classical pyroptosis signalling pathways, thereby endowing an antipyroptotic phenotype and promoting TKI resistance in lung cancer. Our findings provide a new mechanism for lncRNA-mediated TKI resistance from the new perspective of pyroptosis via simultaneous regulation of histone methylation and RNA methylation. The pivotal role of LINC00969 gives it the potential to be a novel biomarker and therapeutic target for overcoming EGFR-TKI resistance in lung cancer.

## Introduction

Lung cancer is one of the leading causes of cancer-related death and most common malignant tumours worldwide [1]. Nearly 85% of lung cancer cases are non-small cell lung cancer (NSCLC) [2]. Epidermal growth factor receptor-tyrosine kinase inhibitors (EGFR-TKI) have previously been demonstrated to be effective against advanced EGFR-mutant NSCLC [3–6]. Unfortunately, acquired resistance still develops in patients after EGFR-TKIs treatment, leading to tumour recurrence and metastasis. Therefore, it is worth further studying the mechanism of EGFR-TKIs resistance and exploring new methods to reverse resistance.

Long noncoding RNAs (lncRNAs) are defined as RNA transcripts that are longer than 200 nt and have limited or no protein-coding capacity [7]. A number of studies have indicated that lncRNAs could play a crucial role in modulating gene expression via multiple mechanisms, such as epigenetic, transcriptional and posttranscriptional mechanisms [7]. Increasing evidence suggests that lncRNAs are closely related to tumorigenesis [8, 9]. Our previous studies also confirmed that lncRNAs can play important regulatory roles in tumorigenesis [10–13]. In addition, lncRNAs play important roles in drug resistance, including resistance to chemotherapies and targeted therapies, in cancer [14–17]. Thus, lncRNAs are closely linked to tumour TKI resistance. However, the detailed mechanisms underlying the roles of lncRNAs in TKI resistance have not been well characterized.

Epigenetic modifications, such as DNA methylation and histone modifications, are crucial for gene regulation and tumorigenesis [18]. In addition, recent studies have indicated that RNA modifications, an important mechanism of epigenetic regulation, are also involved in gene regulation, leading to tumorigenesis and drug resistance. Among these modifications, the posttranscriptional modification of N6-methyladenosine (m6A) RNA methylation is one of the most prevalent [19]. Moreover, aberrant m6A modification is also closely associated with tumorigenesis and drug resistance [19–22]. Numerous studies have indicated that lncRNAs can be involved in epigenetic regulation. However, the roles of lncRNAs that can simultaneously mediate both histone methylation and RNA methylation are still unclear.

Pyroptosis is a newly discovered form of programmed cell death. Pyroptosis can activate inflammasomes via the classical pyroptosis pathway or the nonclassical pyroptosis pathway [23]. Activation of the NOD-like receptor pyrin domain-containing protein 3 (NLRP3) inflammasome results in the conversion of pro-caspase-1 into cleaved caspase-1, and separates the N- and C-termini of Gasdermin D (GSDMD) to trigger the classical pyroptosis pathway [23, 24]. In addition, pyroptosis can activate GSDMD cleavage via the noncanonical inflammasome pathway via caspase-4/5/11, intracellular LPS receptors, rather than with via activation of caspase-1 [24, 25]. There is evidence that pyroptosis is closely related to a variety of biological processes, including tumour malignancy [26–28]. For instance, caspase-1/GSDMD mediated pyroptosis is connected with Taxol resistance in nasopharyngeal carcinoma [26]. MiR-155-5p can enhance susceptibility of breast cancer to cetuximab by activating caspase-1-dependent pyroptosis [28]. No research has been published on the association between pyroptosis and TKI resistance in lung cancer. The dialogue mechanism underlying the crosstalk among lncRNA-mediated epigenetic modifications, pyroptosis and TKI resistance still needs to be further elucidated.

Based on the findings of the present study, LINC00969 can regulate resistance to gefitinib in vitro and in vivo. Gain of H3K4me1 and H3K27Ac can lead to the activation of LINC00969. LINC00969 binds to both EZH2 and METTL3, transcriptionally regulates the level of H3K27me3 in the NLRP3 promoter region, and posttranscriptionally modifies the m6A level in NLRP3 in an m6A-YTHDF2-dependent manner, thus epigenetically repressing NLRP3 expression to suppress the activation of the NLRP3/caspase-1/GSDMD-related classical pyroptosis signalling pathway, endowing an antipyroptotic phenotype and promoting TKI resistance in lung cancer. These findings provide a novel mechanism of lncRNA-mediated TKI resistance from the new perspective of pyroptosis, via simultaneous regulation of histone methylation and RNA methylation. The pivotal role of LINC00969 gives it the potential to be a novel biomarker and therapeutic target for overcoming EGFR-TKI resistance in lung cancer.

## Results

### Bioinformatics analyses of RNA expression profiling data show that histone modifications activate the expression of lncRNA LINC00969, which is upregulated in TKI-resistant lung cancer

**Fig. 1:**
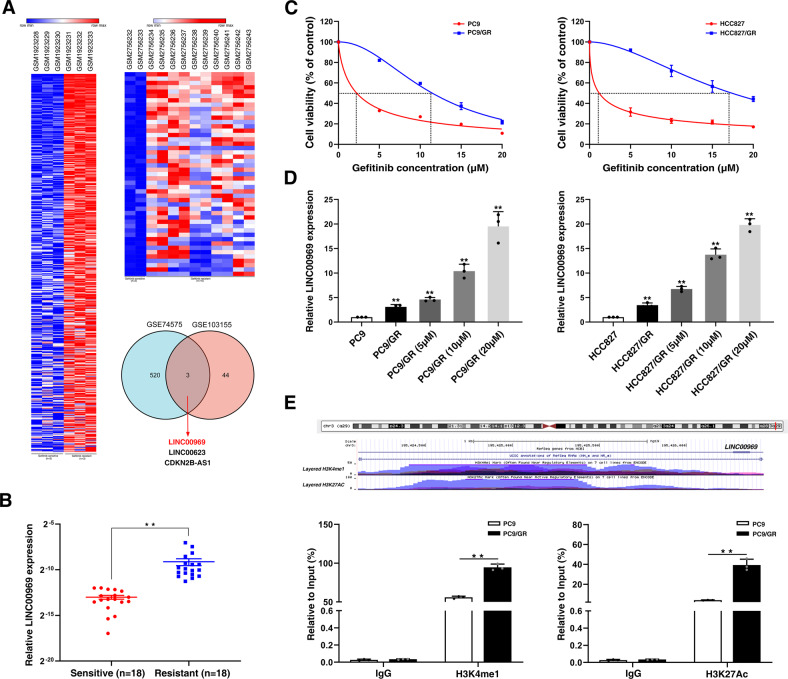
Bioinformatics analyses of RNA expression profiling data show that histone modifications activate the expression of lncRNA LINC00969, which is upregulated in TKI-resistant lung cancer. **A** Relative expression of LINC00969 in gefitinib resistant cells compared with gefitinib sensitive cells in the GSE74575 and GSE103155 datasets. **B** LINC00969 expression levels were analysed in gefitinib-sensitive and gefitinib-resistant NSCLC tissues (sensitive = 18, resistant = 18). **C** IC50 values of gefitinib in resistant cells and their respective parental cells were examined by MTT assays. **D** Quantitative real-time PCR analysis of LINC00969 expression in PC9, PC9/GR, HCC827 and HCC827/GR cells cultured with different concentrations of gefitinib. **E** The UCSC Genome Bioinformatics Site (http://genome.ucsc.edu/) indicated that H3K4me1 and H3K27Ac were highly enriched at regulatory elements of LINC00969. ChIP assays showed the levels of H3K4me1 and H3K27Ac in the promoter of LINC00969 in PC9/GR cells. Three independent experiments were performed. **P < 0.01.

To identify lncRNAs with aberrant expression that are involved in mediating TKI resistance, raw microarray data were downloaded from GEO datasets, including GSE74575 (*n* = 6) and GSE103155 (*n* = 12). GSE74575 contained 3 gefitinib sensitive cells samples (HCC827) and 3 gefitinib resistant cells samples (HCC827/GR). GSE103155 contained 2 gefitinib sensitive cells samples (PC9) and 10 gefitinib resistant cells samples (PC9/GR). We screened for lncRNAs that were activated in TKI-resistant lung cancer (fold change ≥ 2). Then, we found that three lncRNAs (LINC00969, LINC00623, and CDKN2B-AS1) were upregulated in gefitinib-resistant NSCLC cells in both GEO datasets (Fig. 1A). A previous study indicated that LINC00623 plays important roles in regulating the progression hormone-related cancers, including lung cancer, prostate cancer and breast cancer [29]. CDKN2B-AS1 can promote tumorigenesis and chemoresistance [30, 31]. However, there have been no relevant studies to investigate the role of LINC00969 in drug resistance and tumorigenesis. To verify the expression of LINC00969 in gefitinib resistant lung cancer, we collected 36 tissue specimens from patients with advanced NSCLC harbour EGFR exon 19 deletion (Exon 19 Del) or a point mutation in exon 21 (Exon 21 L858R). The patients were divided into two groups: those who never received gefitinib (sensitive group) and those who developed resistance to gefitinib (resistant group). The results of qRT-PCR analysis indicated that the expression of LINC00969 was markedly elevated in the resistant group (Fig. 1B). To confirm the gefitinib resistance characteristics of PC9/GR and HCC827/GR cells, we compared the inhibitory concentrations of gefitinib that resulted in a 50% reduction in cell viability between parental (PC9 and HCC827) and resistant cells (Fig. 1C). As shown in Fig. 1D, we examined the expression of LINC00969 in the paired cell lines, and the differences between gefitinib sensitive cell lines (PC9, HCC827) and gefitinib resistant cell lines (PC9/GR, HCC827/GR) were increased with increasing of gefitinib concentration.

To validate the mechanism of high LINC00969 expression in gefitinib-resistant NSCLC, we first found high enrichment and overlap of H3K4me1 and H3K27Ac at the regulatory elements of LINC00969 utilizing the UCSC Genome Bioinformatics Site (http://genome.ucsc.edu/) (Fig. 1E). In chromatin immunoprecipitation (ChIP) assays, gefitinib-resistant NSCLC cells (PC9/GR) was exhibited increases in H3K4me1 and H3K27Ac at regulatory elements of LINC00969 compared with the levels in gefitinib-sensitive NSCLC cells (PC9) (Fig. 1E). These findings revealed that LINC00969 expression is frequently increased in gefitinib-resistant NSCLC. The higher levels of H3K4me1 and H3K27Ac at regulatory elements of LINC00969 might partially explain the dysregulation of LINC00969 expression in gefitinib resistance.

### LINC00969 promotes resistance to gefitinib in vitro

**Fig. 2:**
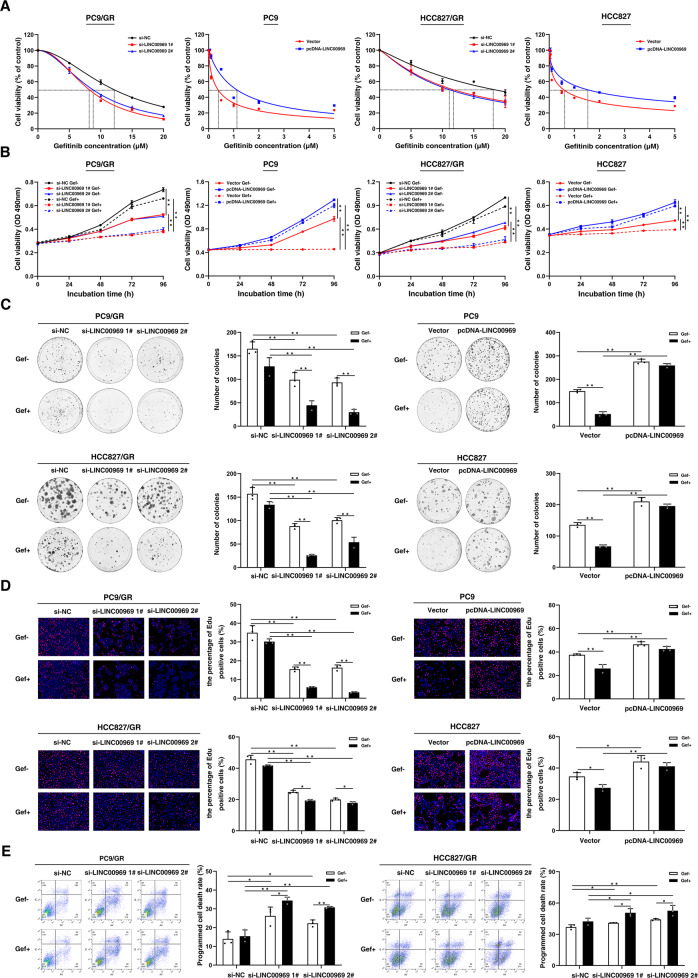
LINC00969 promotes resistance to gefitinib in vitro. **A** MTT assays were performed to determine the IC50 of gefitinib in gefitinib-resistant cells after knockdown of LINC00969, and gefitinib sensitive cells after overexpression of LINC00969. **B**–**D** MTT assays, colony formation assays and EdU assays were performed to determine the cell proliferation potential after LINC00969 expression was changed. **E** Flow cytometric analyses of PC9/GR and HCC827/GR cells were performed 48 h after transfection. LR, early programmed cell death cells. UR, terminal programmed cell death cells. All bars show the mean ± SD of three independent experiments. *P < 0.05, **P < 0.01.

To investigate the potential role of LINC00969 in gefitinib-resistant NSCLC cells, small interfering RNA (siRNA)-mediated knockdown was used in conjunction with plasmid-mediated overexpression to exogenously change LINC00969 expression. qRT-PCR analysis showed that LINC00969 expression was markedly reduced after siRNA treatment (Fig. S1A). Moreover, a LINC00969 overexpression vector was transfected into parental PC9 and HCC827 cells to increase the LINC00969 expression level (Fig. S1B). MTT assays showed that knockdown of LINC00969 significantly decreased the IC50 value of gefitinib in PC9/GR and HCC827/GR cells, and that LINC00969 overexpression induced gefitinib resistance in PC9 and HCC827 cells (Fig. 2A). Moreover, downregulation of LINC00969 expression not only inhibited the proliferation but also restored the gefitinib sensitivity of PC9/GR and HCC827/GR cells. In contrast, LINC00969 overexpression promoted cell proliferation regardless of gefitinib treatment (Fig. 2B). Based on the colony formation assays, clonogenic survival decreased significantly after siRNA knockdown of LINC00969, particularly in the gefitinib group. Additionally, overexpression of LINC00969 led to an increase in the number of clones formed regardless of gefitinib treatment (Fig. 2C). Similarly, EdU assays revealed that either knockdown or overexpression of LINC00969 significantly impacted the proliferation of gefitinib-resistant and gefitinib-sensitive cells (Fig. 2D). Flow cytometric analysis was used to determine whether LINC00969 is involved in the regulation of programmed cell death, including apoptosis, pyroptosis, and so on. In comparison with the control cells, gefitinib-resistant LINC00969 knockdown cells exhibited an increase in the programmed cell death rate for cells, and the programmed cell death rate of LINC00969 knockdown cells treated with gefitinib was further increased (Fig. 2E). Overall, these results suggested that LINC00969 could promote resistance to gefitinib in vitro.

### LINC00969 regulates gefitinib resistance by inhibiting the NLRP3-induced classical pyroptosis pathway

**Fig. 3:**
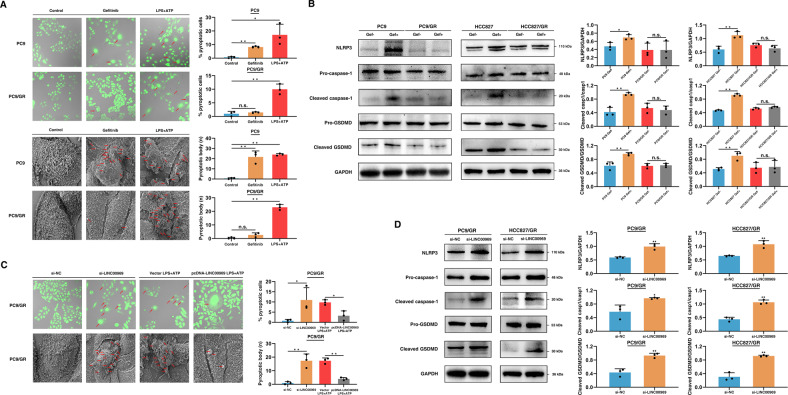
LINC00969 regulates gefitinib resistance by inhibiting the NLRP3-induced classical pyroptosis pathway. **A** PC9 and PC9/GR cell morphology was observed using confocal microscopy and electron microscopy after treatment with gefitinib or LPS/ATP for 48 h. **B** Western blot analysis of NLRP3, pro-caspase-1, cleaved caspase-1, pro-GSDMD, and cleaved GSDMD protein levels after treatment with or without gefitinib. **C** After 48 h of transfection with siRNA or the overexpression plasmid, the morphology of PC9/GR cells was observed after treatment with gefitinib. **D** Western blot analysis of NLRP3, pro-caspase-1, cleaved caspase-1, pro-GSDMD, and cleaved GSDMD protein levels in gefitinib-treated resistant cells transfected with si-NC or si-LINC00969 after treated with gefitinib. Three independent experiments were performed. *P < 0.05, **P < 0.01; n.s. not significant.

Pyroptosis is a lytic and inflammatory type of programmed cell death that is usually triggered by inflammasomes and executed by gasdermin proteins [32]. Previous studies have shown that pyroptosis is associated with tumorigenesis and drug resistance [25–27]. For example, the caspase-1/GSDMD-mediated pyroptosis pathway plays a crucial role in Taxol resistance in nasopharyngeal carcinoma [26]. Pyroptosis is considered an important factor in the susceptibility of breast cancer to cetuximab [28]. However, it remains unclear how pyroptosis contributes to TKI resistance. Therefore, we speculated that pyroptosis may also have a unique function in TKI resistance. To test this hypothesis, PC9 and PC9/GR cells were treated with gefitinib. In addition, cells were treated with lipopolysaccharide (LPS) and ATP were considered positive pyroptosis controls [33]. Then, these treated cells were used for analysis of the pyroptosis phenotype. As shown in Fig. 3A, PC9 cells treated with gefitinib exhibited pore formation in the plasma membrane, swelling, and even lysis, as determined by confocal microscopy (calcein staining) and electron microscopy (Fig. 3A). Nevertheless, PC9/GR cells did not exhibit obvious morphological changes (Fig. 3A). These results suggest that in TKI-resistant cells exhibit a pyroptosis resistance phenotype.

There is increasing evidence that NLRP3-related pyroptosis is crucial for the progression of cancer, as well as tumour drug resistance [28, 34–36]. NLRP3 inflammasome formation is a key event in the activation of the classical pyroptosis pathway [37]. The NLRP3 inflammasome converts pro-caspase-1 to cleaved caspase-1 and then cleaves GSDMD, leading to pyroptosis [23]. Based on this, we further elucidated whether gefitinib exerts a significant effect by promoting the activity of the NLRP3-induced classical pyroptosis pathway. Then we evaluated the expression of related key genes in the NLRP3-induced classical pyroptosis pathway in resistant and sensitive cells. As expected, after treatment with gefitinib, the protein levels of NLRP3, cleaved-caspase-1 and cleaved-GSDMD were increased in sensitive cells compared with resistant cells (Fig. 3B). These findings suggest that gefitinib induces pyroptosis by activating the NLRP3/caspase-1/GSDMD pathway in EGFR-positive NSCLC cells. In addition, inhibition of the NLRP3-induced classical pyroptosis pathway may be one of the major gefitinib resistance mechanisms.

We next sought to obtain a deeper understanding of LINC00969 in the anti-pyroptotic phenotype of gefitinib-resistant NSCLC cells. After treatment with gefitinib, knockdown of LINC00969 induced pyroptosis in PC9/GR cells, as shown by confocal microscopy and electron microscopy (Fig. 3C). Furthermore, overexpression of LINC00969 reversed ATP/LPS-induced pyroptosis in PC9/GR cells (Fig. 3C). The NLRP3, cleaved-caspase-1 and cleaved-GSDMD protein levels were increased in both PC9/GR and HCC827/GR cells following knockdown of LINC00969 (Fig. 3D). It was concluded from these results that LINC00969 can suppress the NLRP3-induced classical pyroptosis pathway to promote gefitinib resistance in NSCLC.

### LINC00969 interacts with EZH2 and METTL3

A large number of lncRNAs have been demonstrated to interact with RNA-binding proteins (RBPs), thus epigenetically regulating gene expression, especially in cancer [38–40]. There is evidence that approximately 20% of human noncoding RNAs are functionally associated with polycomb repressive complex 2 (PRC2) [41]. PRC2 is able to catalyse the trimethylation of lysine 27 in histone 3 (H3K27me3), which can epigenetically regulate gene transcription [42]. Enhancer of zeste homologue 2 (EZH2), a core subunit of PRC2, plays a vital role in tumorigenesis and drug resistance [41]. The RNA methyltransferase methyltransferase-like 3 (METTL3), a key m6A “writer” that mediates N6-methyladenosine (m6A) RNA methylation, is implicated in mRNA processing, localization, decay, especially, posttranscriptional regulation [43–46]. Previous studies have found that METTL3 regulates tumour cell proliferation and drug resistance by affecting the m6A levels of target RNAs [19, 43, 47, 48]. Thus, we hypothesized that LINC00969 can control gene expression at both the transcriptional and posttranscriptional levels.

**Fig. 4:**
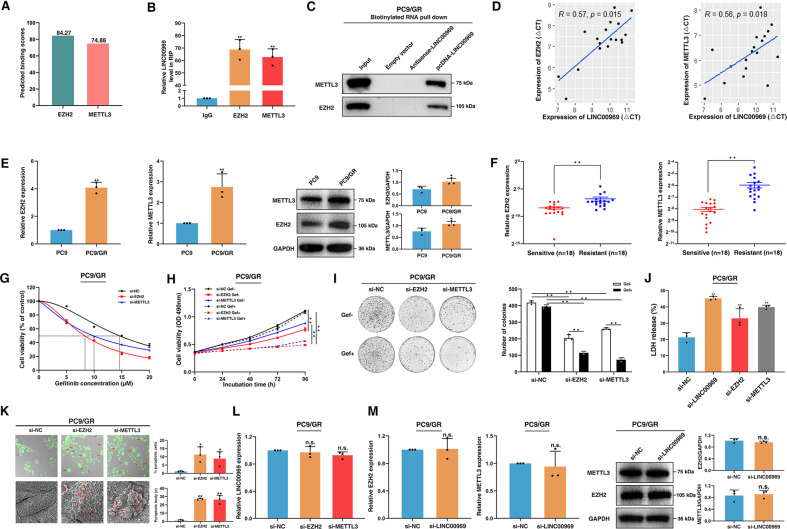
LINC00969 functions by simultaneously interacting with EZH2 and METTL3. **A** LINC00969 and RBPs can interact, based on the results of bioinformatics analysis. **B** RIP assays were conducted in PC9/GR cells, and qRT-PCR was performed on the coprecipitated RNA to analyse LINC00969 expression. **C** RNA pull-down assays and WB analysis demonstrated that LINC00969 bound to EZH2 and METTL3 simultaneously in PC9/GR cells. **D** The correlation between the expression of LINC00969 and the expression of EZH2 and METTL3 was analysed in gefitinib resistant NSCLC tissues. **E** The mRNA and protein levels of EZH2 and METTL3 in PC9 and PC9/GR cells. **F** EZH2 and METTL3 expression levels were analysed in gefitinib-sensitive and gefitinib-resistant NSCLC tissues. **G** MTT assays were used to evaluate the IC50 value of gefitinib in PC9/GR cells transfected with si-EZH2 and si-METTL3. **H**, **I** MTT assays and colony formation assays were performed to test the viability of cells after knockdown of EZH2 and METTL3. **J** An LDH release assay was performed after silencing of LINC00969, EZH2 and METTL3. **K** The morphology of PC9/GR cells transfected with si-EZH2 and si-METTL3 was observed after treatment with gefitinib for 48 h. **L** After knockdown of EZH2 and METTL3, the expression of LINC00969 was evaluated. **M** The mRNA and protein levels of EZH2 and METTL3 after knockdown of LINC00969. Three independent experiments were performed. *P < 0.05, **P < 0.01; n.s. not significant.

The interaction potential of LINC00969 and EZH2/METTL3 has been predicted using bioinformatics approaches (http://bioinfo.bjmu.edu.cn/lncpro/) [49]. As shown in Fig. 4A, LINC00969 was predicted to have a high probability of interaction with EZH2 and METTL3, with a high score. Then, we performed RNA immunoprecipitation (RIP) assays to further evaluate the interactions of LINC00969 with EZH2 and METTL3 in PC9/GR cells (Fig. 4B). According to the results, LINC00969 bound to EZH2 and METTL3. Moreover, the RNA pull-down assay results further confirmed that EZH2 and METTL3 were detectable only in the complexes precipitated with LINC00969, not in the complexes precipitated with either the empty vector or antisense LINC00969, in PC9/GR cells (Fig. 4C). The expression of LINC00969 was positively correlated with the expression of EZH2 and METTL3 in gefitinib-resistant NSCLC tissues (Fig. 4D). In clarification the postulated roles of EZH2 and METTL3 in gefitinib resistance, EZH2 and METTL3 were significantly overexpressed in PC9/GR cells compared to PC9 cells, at both the mRNA and protein levels (Fig. 4E). In addition, gefitinib-resistant tissues exhibited much higher expression of EZH2 and METTL3 than gefitinib-sensitive tissues (Fig. 4F). In PC9/GR cells, inhibition of EZH2 and METTL3 expression prevented cell proliferation and restored gefitinib sensitivity (Fig. 4G–I). We used a lactate dehydrogenase (LDH) assay to determine whether membrane integrity was lost during pyroptosis. LDH release was significantly increased after silencing of LINC00969, EZH2 or METTL3 (Fig. 4J). We next sought to further explore the roles of EZH2 and METTL3 in the pyroptosis of gefitinib-resistant NSCLC cells and found that siRNA-EZH2 and siRNA-METTL3 induced pyroptosis in PC9/GR cells (Fig. 4K). These data indicated that LINC00969, EZH2 or METTL3 are closely related to pyroptosis. Additionally, knockdown of EZH2 or METTL3 had no effect on the expression of LINC00969 (Fig. 4L). Conversely, qRT-PCR and Western blot analyses also showed that knockdown of LINC00969 did not affect EZH2 or METTL3 expression, at either the mRNA or protein level (Fig. 4M). According to these findings, LINC00969 may be involved in the acquisition of gefitinib resistance in NSCLC by regulating other genes by binding to EZH2 and METTL3, in a manner that affects cell pyroptosis.

### LINC00969 regulates the expression of NLRP3 at the transcriptional and posttranscriptional levels, thus suppressing the classical pyroptosis pathway to promote gefitinib resistance

**Fig. 5:**
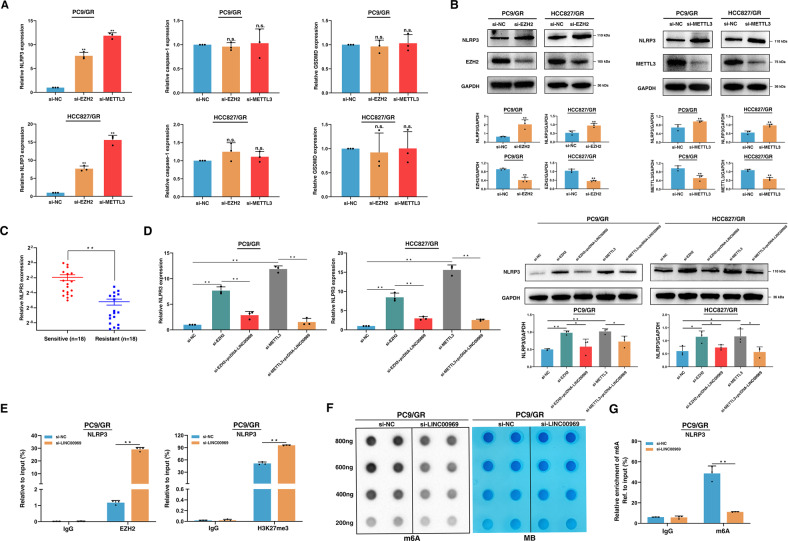
LINC00969 interacts with EZH2 and METTL3, transcriptionally regulates the level of H3K27me3 in the NLRP3 promoter region, and posttranscriptionally modifies the m6A level of NLRP3, thus suppressing the classical pyroptosis pathway to promote gefitinib resistance. **A** The mRNA levels of NLRP3, caspase-1, and GSDMD in gefitinib resistant cells transfected with si-EZH2 or si-METTL3 after gefitinib treatment. **B** The protein level of NLRP3 in gefitinib resistant cells transfected with si-EZH2 or si-METTL3 after gefitinib treatment. **C** NLRP3 expression levels were analysed in gefitinib-sensitive and gefitinib-resistant NSCLC tissues. **D** Measurement of the NLRP3 level after LINC00969 overexpression in the gefitinib-resistant cells transfected with si-EZH2 or si-METTL3. **E** ChIP-qPCR of H3K27me3 and EZH2 in the NLRP3 promoter regions in PC9/GR cells after following treatment targeting si-NC and si-LINC00969. Antibody enrichment was quantified based on the amount of input DNA. IgG was used as the negative control. **F** M6A dot blot analysis of PC9/GR cells with LINC00969 knockdown. Methyl blue (MB) stain was used as a loading control. **G** qRT-PCR was used to measure the m6A level in NLRP3 in control and LINC00969 knockdown cells after immunoprecipitation. Three independent experiments were performed. *P < 0.05, **P < 0.01; n.s. not significant.

We previously discovered that LINC00969 can suppress the NLRP3-induced classical pyroptosis pathway to promote gefitinib resistance. Next, we explored whether LINC00969 regulates key genes associated with the classical pyroptosis pathway in an EZH2 and METTL3-dependent manner. NLRP3 was upregulated after silencing of EZH2 or METTL3, however, knockdown of EZH2 or METTL3 did not affect the expression of caspase-1 and GSDMD, in either PC9/GR or HCC827/GR cells (Fig. 5A, B, S1C and S1D). In addition, we found that NLRP3 expression was indeed reduced in gefitinib-resistant tissues (Fig. 5C). Moreover, we transfected EZH2 and METTL3 knockout cells with the LINC00969 overexpression, respectively. Overexpression of LINC00969 partially reversed the decrease in NLRP3 expression induced by knockdown of EZH2 or METTL3 (Fig. 5D).

To obtain further direct evidence, our next step was to perform chromatin immunoprecipitation (ChIP) assays. The results of these assays revealed that knockdown of LINC00969 decreased EZH2 binding and H3K27me3 modification throughout the promoter of NLRP3. Thus, we confirmed that LINC00969 can interact with the EZH2 protein to epigenetically suppress the expression of NLRP3 at the transcriptional level (Fig. 5E). Dot blot analysis of the m6A level in PC9/GR cells indicated that LINC00969 knockdown significantly decreased the levels of m6A (Fig. 5F). This finding demonstrated that LINC00969 can function by regulating the abundance of the m6A during the acquisition of gefitinib resistance. Moreover, the results of MeRIP assays verified the reduction in the m6A level of NLRP3 after LINC00969 knockdown (Fig. 5G). These results revealed that LINC00969 regulates the expression of NLRP3 in an m6A modification-dependent manner by binding to METTL3.

**Fig. 6:**
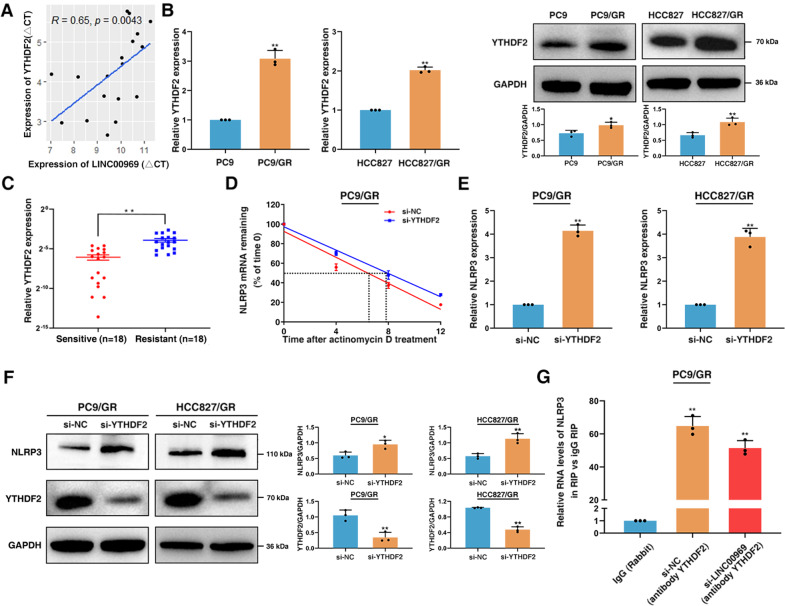
LINC00969 regulates the expression of NLRP3 in an m6A-YTHDF2-dependent manner. **A** Correlation analysis between the expression of LINC00969 and YTHDF2 in gefitinib-resistant NSCLC samples. **B** YTHDF2 mRNA and protein levels in gefitinib sensitive and resistant NSCLC cells. **C** YTHDF2 expression levels were analysed in gefitinib-sensitive and gefitinib-resistant NSCLC tissues. **D** Half-life of NLRP3 mRNA after knockdown YTHDF2. **E** qRT-PCR was performed to analyse the NLRP3 expression after transfection with si-YTHDF2. **F** The protein levels were analysed after knockdown of NLRP3 in PC9/GR and HCC827/GR cells. **G** A RIP experiment for YTHDF2 was conducted in PC9/GR cells after transfection of si-NC and si-LINC00969, and qRT-PCR for NLRP3 was performed on the coprecipitated RNA. Three independent experiments were performed. *P < 0.05, **P < 0.01.

In addition, we further investigated how METTL3 affects the m6A level of NLRP3. Various m6A“reader” proteins, including YTHDF1, YTHDF2 and YTHDF3, can recognize m6A-modified sites and exploit them for different functions. In particular, YTHDF2 can regulate cancer progression by binding to m6A-modified sites to mediate mRNA degradation [45, 50]. Moreover, our analysis revealed a positive expression correlation between LINC00969 and YTHDF2 expression in gefitinib-resistant tissues (Fig. 6A). YTHDF2 was significantly overexpressed in both PC9/GR and HCC827/GR cells at both the mRNA and protein levels (Fig. 6B). Moreover, YTHDF2 expression was increased in gefitinib-resistant tissues (Fig. 6C). In addition, the half-life of NLRP3 transcripts was increased after silencing of YTHDF2 in PC9/GR cells (Fig. 6D). Measurement of mRNA and protein levels indicated NLRP3 downregulation after YTHDF2 knockdown (Figs. 6E, F and S1E). In addition, the RIP assay showed that YTHDF2 can bind to NLRP3 mRNA (Fig. 6G). After silencing of LINC00969, the amount of YTHDF2 bound to NLRP3 mRNA was significantly reduced (Fig. 6G). These results demonstrated that LINC00969 can interact with METTL3 and posttranscriptionally regulate NLRP3 expression in an m6A-YTHDF2-dependent manner. Together, these findings indicate that LINC00969 simultaneously interacts with EZH2 and METTL3, thereby regulating the expression of NLRP3 at the transcriptional and posttranscriptional levels, thus suppressing the NLRP3-induced classical pyroptosis pathway to promote gefitinib resistance in NSCLC.

### LINC00969 contributes to NSCLC tumorigenesis

**Fig. 7:**
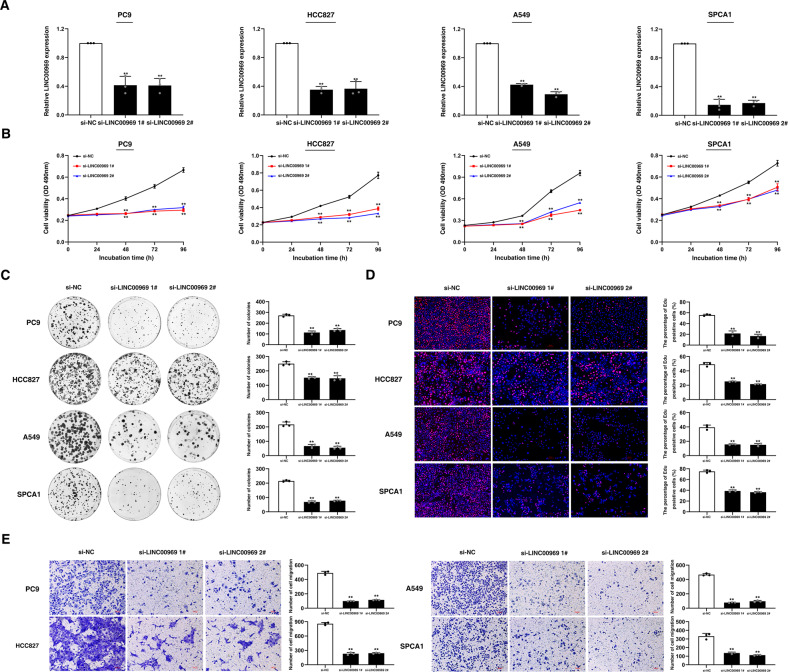
LINC00969 contributes to NSCLC tumorigenesis. **A** qRT-PCR analysis of LINC00969 expression in NSCLC cells (PC9, HCC827, A549 and SPCA1) transfected with siRNAs. **B**–**D** MTT, colony formation and EDU assays were performed to assess the proliferation of NSCLC cells after transfection. **E** After transfection, NSCLC cells were subjected to Transwell assays to evaluate changes in the migration capability. All bars show the mean ± SD of three independent experiments. **P < 0.01.

To investigate the role of LINC00969 in NSCLC tumorigenesis, the expression of LINC00969 was knocked down in NSCLC cell lines (PC9, HCC827, A549 and SPCA1) (Fig. 7A). As shown in Fig. 7B–D, cell proliferation was markedly decreased following knockdown of LINC00969. In addition, transwell assays showed that knockdown of LINC00969 obviously suppressed the migration of NSCLC cells (PC9, HCC827, A549 and SPCA1) (Fig. 7E).

### LINC00969 regulates resistance to gefitinib in vivo

**Fig. 8:**
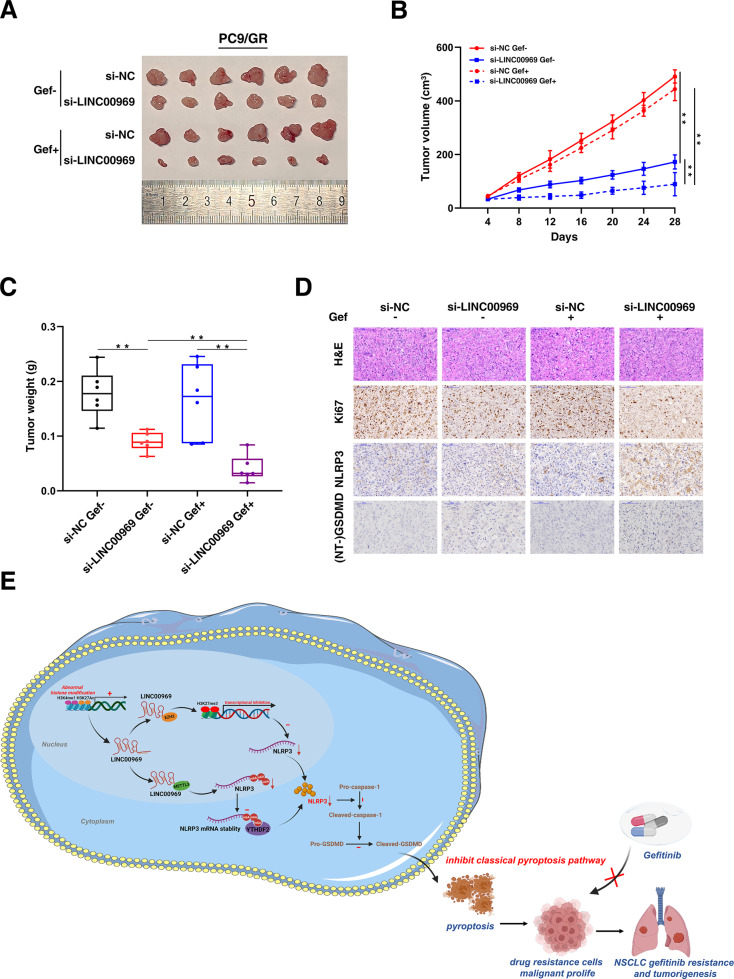
LINC00969 regulates resistance to gefitinib in vivo. PC9/GR cells were injected into 12 nude mice. Once tumours were palpable, and intratumorally administered with in vivo-optimized si-LINC00969 and control (5 nmol per injection) once every 4 days. In addition, Gefitinib (25.0 mg/kg) or the corresponding control treatment was administered to the mice once a day. **A** Pictures of the tumours are shown and were compared among the groups. **B** Every 4 days, the tumour volume was calculated. **C** Tumour weights were calculated and presented as the mean tumour weight ± S.D. (standard deviation) values. **D** The tumour sections were subjected to H&E staining and immunohistochemical for Ki67, NLRP3 and (NT-)GSDMD. **E** Proposed model in which LINC00969 mediates gefitinib resistance and tumorigenesis in NSCLC. All bars show the mean ± SD of three independent experiments. **P < 0.01.

To further determine the role of LINC00969 in NSCLC with EGFR mutation and gefitinib resistance in vivo, the same number of PC9/GR cells were injected into nude mice. Once tumours were palpable, and intratumorally administered with in vivo-optimized si-LINC00969 and control (5 nmol per injection) once every 4 days. In addition, Gefitinib (25.0 mg/kg) or the corresponding control treatment was administered to the mice once a day. Until 4 weeks after cell injection, the tumours were substantially smaller in the mice injected with si-LINC00969 group, and the tumour size was further decreased with gefitinib treatment (Fig. 8A). The tumour volume and weight were markedly lower than those in the control-treated group (Figs. 8B and C). As demonstrated by immunohistochemical (IHC) staining, the expression of the proliferation marker Ki67 was decreased after silencing of LINC00969 regardless of gefitinib treatment (Fig. 8D). Furthermore, NLRP3 and (NT-)GSDMD was identified as the major downstream mediator of pyroptosis [37, 51]. Significantly higher expression of NLRP3 and (NT-)GSDMD were observed in knockdown-LINC00969 derived tumours treated with gefitinib (Fig. 8D). These findings complemented that LINC00969 promotes tumour growth and gefitinib resistance by inhibiting the NLRP3-mediated pyroptosis pathway in vivo.

## Discussion

Numerous clinical trials have suggested that EGFR-TKIs, such as gefitinib, are the standard first-line therapy for patients with EGFR mutant NSCLC [5]. However, acquired resistance usually occurs during treatment with EGFR-TKIs [52]. The mechanisms of acquired resistance to EGFR-TKIs are particularly complex. In recent years, it was proven that lncRNAs were closely related to tumorigenesis and drug resistance. For instance, the lncRNA DILA1 promotes cell proliferation and tamoxifen resistance by downregulating CyclinD1 in breast cancer [17]. The lncRNA AFAP1-AS1 regulates chemotherapeutic resistance by activating the RRM2/EGFR/AKT signalling pathway in NSCLC [53]. In our study, we screened lncRNAs and identified the involvement of the lncRNA LINC00969 in TKI resistance in lung cancer. This involvement was verified in TKI resistant cells and tissues. Subsequent assays were conducted in vitro and in vivo to further verify the role of LINC00969 in gefitinib resistance.

LINC00969 is located at chromosome on 3q29. Yu et al. were the first to discover that LINC00969 can promote intervertebral disc degeneration [54]. In contrast, the role of LINC00969 and its molecular mechanisms in tumorigenesis and TKI resistance in lung cancer remain largely unknown. Similar to its presence in protein-coding genes, epigenetic modification of the promoters of lncRNAs has been clarified in many studies. For example, our previous work showed that H3K27 acetylation-mediated activation of the promoter can lead to activation of lncRNA CCAT1 expression in esophageal squamous cell carcinoma [55]. Previous studies have also found that abnormal modifications of H3K27Ac and H3K4me1 could participate in drug resistance by activating lncRNA expression. For example, lncRNA ZNF649-AS1 was induced by H3K27 acetylation in trastuzumab resistance of breast cancer. The enrichment level of H3K27Ac was increased in SKBR-3-TR (trastuzumab resistant) and BT474-TR cells compared to their parental sensitive cells [56]. LncRNA DLX6-AS1 expression was induced by H3K4me1 in cisplatin resistance of LUSC (lung squamous cell carcinoma). The enrichment level of H3K4me1 was increased in SK-MES-1-resistance compared to parental sensitive cells [57]. Our data showed that gain of H3K4me1 and H3K27Ac can induce the expression of LINC00969 in gefitinib-resistant NSCLC cells, indicating that the involvement of abnormal histone modification in drug resistance is partly achieved through the regulation of LINC00969.

In addition, we explored the mechanism of action of LINC00969 in the process of gefitinib resistance. There is increasing evidence that lncRNAs interact with many RBPs to perform their functions [58, 59]. A core component of the PRC2 complex that is capable of installing H3K27me3, EZH2, has critical roles in tumorigenesis and drug resistance [11, 60]. A significant increase in EZH2 expression was found in NSCLC cells resistant to gefitinib. METTL3 is an essential component of the complex involved in m6A methylation [61]. METTL3 was reported to have a critical m6A-dependent role in facilitating cancer progression and metastasis by an m6A dependent manner [19, 62]. Our previous study also demonstrated that METTL3 could promote resistance to gefitinib in NSCLC [63]. According to the current study, LINC00969 promotes gefitinib resistance in lung cancer by simultaneously binding to EZH2 and METTL3.

Pyroptosis, an entirely new form of programmed cell death, is caused by inflammasome-mediated cell swelling and even membrane rupture [24]. Previously, it was shown that pyroptosis plays a crucial role in tumorigenesis and drug resistance [26, 28, 64]. LncRNAs have the potential to play important roles in the treatment of a variety of diseases by regulating cell pyroptosis [65–67]. For example, lncRNA Kcnq1ot1 regulates pyroptosis, in turn activating fibrosis, in diabetic cardiomyopathy [67]. The lncRNA MEG3 promotes cerebral ischemia-reperfusion injury by increasing pyroptosis [66]. However, the function and mechanism of lncRNAs in the regulation of drug resistance-related pyroptosis to facilitate TKI resistance in lung cancer remain unclear. Moreover, the relationships among histone methylation, RNA methylation and pyroptosis are also unclear. Our data showed that NLRP3 was activated after LINC00969 knockdown. NLRP3 has been reported to be a crucial component of the classical pyroptosis pathway in many studies [24]. We confirmed that knockdown of LINC00969 prevented EZH2 binding to the NLRP3 promoter and inhibited H3K27 trimethylation, in turn inhibiting the NLRP3-mediated pyroptosis pathway, thus contributing to gefitinib resistance in NSCLC. In addition, LINC00969 can interact with METTL3 to regulate the expression of NLRP3 in an m6A-YTHDF2-dependent manner. LINC00969 regulates the expression of NLRP3 at the transcriptional and posttranscriptional levels by simultaneously regulating two epigenetic modifications, i.e., histone methylation and RNA methylation, thereby regulating pyroptosis in drug-resistant cells and promoting drug resistance.

Our study suggests that LINC00969 can regulate the expression of NLRP3 at the transcriptional and posttranscriptional levels, thus suppressing the classical pyroptosis pathway to promote gefitinib resistance. In spite of this, our study has several limitations. First of all, small sample size of patients is the drawback of our study. It would be best to collect more patient tissue samples and integrate the data into our studies. And we noted that (NT-)GSDMD significantly decreased in knockdown-LINC00969 derived tumors treated with gefitinib by immunohistochemistry (Fig. 8D). The results of our study are different from previous studies. Tonnus et al. found relatively high abundance of expression of (NT-)GSDMD in kidney tissues of acute kidney injury patients [51]. The barely (NT-)GSDMD cells in cancer tissues may be due to the specificity of disease and tissue.

In summary, this is the first study to prove that LINC00969 regulates TKI resistance via this mechanism. We also demonstrate the functional role of LINC00969 in lung tumorigenesis. LINC00969 acts as a mediator and regulator of interactions among histone methylation, RNA methylation, and pyroptosis, and regulates the acquisition of an antipyroptotic phenotype in TKI resistance. LINC00969-mediated regulation of TKI resistance could enrich the knowledge of tumour resistance, and LINC00969 has great prospects as be a prognostic indicator and a potential therapeutic target for overcoming EGFR-TKI resistance in NSCLC (Fig. 8E).

## Materials and methods

### Tissue collection and ethics statement

A total of 36 patients with advanced NSCLC who had either EGFR exon 19 deletion (Exon 19 Del) or exon 21 L858R mutation (Exon 21 L858R) were enrolled in this study. The gefitinib-sensitive group contained 18 patients, and the patients in the other groups were enrolled after the development of acquired EGFR-TKI resistance. All patients provided written informed consent following approval of the study by the Research Ethics Committee of the First Affiliated Hospital of Nanjing Medical University.

### Cell culture

PC9 (EGFR Exon 19 Del), HCC827 (EGFR Exon 19 Del), A549, and SPCA1 cells were obtained from the Institute of Biochemistry and Cell Biology at the Chinese Academy of Sciences (Shanghai, China). The PC9/GR and HCC827/GR cell lines were established in our laboratory by stepwise escalation of the concentration of gefitinib over a 6-month period. All cell lines were identified by short tandem repeat (STR) analysis (Biowing, Shanghai, China) and tested for mycoplasma contamination. PC9, PC9/GR and SPCA1 cells were cultured in DMEM medium. HCC827, HCC827/GR and A549 cells were cultured in RPMI-1640 medium. To both DMEM and RPMI-1640 medium, 10% foetal bovine serum (FBS) and antibiotics (100 U/mL penicillin and 100 mg/mL streptomycin) were added. All cells were placed in a 37 °C humidified incubator with 5% CO_2_.

### RNA extraction and qRT-PCR analyses

Cells were harvested with TRIzol (Invitrogen) reagent to isolate total RNA. A PrimerScript RT Reagent Kit (Takara) was used to reverse transcribe RNA (1.0 μg) to cDNA by quantitative reverse transcription PCR (qRT-PCR). Real-time PCR analysis was conducted with SYBR Green (Takara). The expression of glyceraldehyde-3-phosphate dehydrogenase (GAPDH) was used to normalize expression data. The specific primers were listed in Table S1. Each assay was replicated three times.

### Cell transfection

Transfection of NSCLC cells with the specific siRNA and negative control siRNA (si-NC) was performed using Lipofectamine 2000 (Invitrogen), in accordance with the manufacturer’s instructions. The LINC00969 siRNA was obtained from Invitrogen along with si-NC. The nucleotide sequences of the siRNAs were shown in Table S1. X-tremeGENE™ HP DNA Transfection Reagent (Roche) was used to transfect plasmids into PC9 and HCC827 cells. At 48 h following transfection, cells were harvested and analysed by qRT-PCR or western blotting.

### Cell proliferation assay

The proliferation of cells was measured with an MTT kit (Sigma) following the manufacturer’s protocol. The colony formation assay was performed by seeding transfected cells into six well plates (1500 cells per well) and culturing them for two weeks. Following seeding, the medium containing drugs was replaced every 4 days. The colonies were fixed with methanol and then stained with 0.1% crystal violet in PBS for 15 min. By counting the stained colonies, colony formation was quantified. A kit for labelling/detecting proliferating cells (RiboBio) was used in accordance with the manufacturer’s instructions. Confocal laser scanning microscopy was used to determine the percentage of EdU-positive cells. The experiment included three replicates.

### Flow cytometric analysis

Following transfection for 48 h, cells were harvested. In accordance with the instructions given by the manufacturer, a double staining was performed using fluorescein isothiocyanate (FITC)-Annexin V and propidium iodide with FITC Annexin V programmed cell death Detection Kit (BD Biosciences). A flow cytometer (FACScan; BD Biosciences) equipped with Cell Quest software (BD Biosciences) was used to analyse the cells. Cell viability was examined by separating the viable, dead, early programmed death and programmed death cells. A comparison was made between the numbers of early programmed death cells and control cells.

### Calcein-AM assay

Cells were seeded into in 35 mm confocal dishes. Following the manufacturer’s instructions, the cells were stained with calcein-AM for 20 min. Then, the stained cells were viewed using confocal microscopy (Olympus).

### Electron microscopy imaging

Fixation with 2.5% glutaraldehyde for 3 h followed by dehydration in a graded ethanol series (30%, 50%, 70%, 90%, and 100%) and drying in tertiary butanol was employed. After drying in a silica gel vacuum desiccator, the sample was adhered to the sample stage with double-sided conductive tape and examined under a transmission electron microscope (JEOL JSM-7900F).

### Cell migration assays

Migration assays were performed using polycarbonate membranes (Corning) with 8 mm pores in 24-well Transwell plates. The transfected cells were seeded in the upper compartment in serum-free medium. Afterwards, 10% FBS was added to the lower compartment. After 24 h, methanol and 0.5% crystal violet solution were used to detect the cells that had migrated through the membrane. The migrated cells were imaged and counted using an IX71 inverted microscope (Olympus). Each experiment was repeated three times.

### Animal xenograft tumour model

Nanjing Medical University’s Animal Experimental Ethics Committee approved the animal experiments. To establish xenograft models, five-week-old male mice were orthotopically injected with the same number of the PC9/GR cells. Tumours had developed after 4 days, at which time the xenografted mice were randomly divided into the following two experimental groups (each group with 6 mice): (1) the control group and (2) the gefitinib group. Gefitinib treatment was administered every day at a dose of 25 mg/kg. In addition, the tumours were intratumorally administered with in vivo-optimized si-LINC00969 and control (5 nmol per injection, RiboBio) once every 4 days. Every four days, the mice were weighed, and tumour volume were measured. The tumour volume was calculated as 0.5 × length × width^2^. After 4 weeks of injection, all mice were euthanized and tumour weights were measured. Moreover, the resected tumour were used for immunohistochemical (IHC) staining.

### Chromatin immunoprecipitation (ChIP) assay

We performed ChIP assays with the EZ-ChIP Kit following the instructions provided by the manufacturer (Millipore). The antibodies specific for histone H3, histone H3 acetylated at Lys27 (H3K27Ac, Cat. # ab4729), and H3 monomethylated at Lys4 (H3K4me1, Cat. # ab8895) were purchased from Abcam. The antibodies specific for EZH2 and H3 trimethylated at Lys 27 (H3K27me3, Cat. # ab6002) were purchased from Abcam. Table S1 lists the primer sequences used for ChIP. The immunoprecipitated DNA was quantified by qPCR with SYBR Green Mix (Takara). ChIP data were expressed as percentages of input DNA according to the following equation: 2^[Input Ct− Target Ct]^ × 100 (%).

### RNA immunoprecipitation (RIP) assay

A Magna RIP RNA-Binding Protein Immunoprecipitation Kit (Millipore, USA) was used for RIP assays on PC9/GR cells, as per the instructions provided by the manufacturer. Abcam provided the antibodies used for RIP assays of EZH2 and METTL3.

### In vitro transcription assay and RNA pull down assay

According to the manufacturer’s instructions, translation assays were performed using Invitrogen’s mMESSAGE mMACHINETM T7 Transcription Kit. LINC00969 transcripts were labelled with desthiobiotin using the Pierce RNA 3’ End Desthiobiotinylation Kit (Pierce, Thermo). RNA pull-down was performed with Pierce Magnetic RNA/Protein Pull-Down Kits according to the accompanying instructions (Pierce, Thermo).

### LDH release assay

After cells were treated with siRNA for 48 h, the culture supernatants were collected. According to the manufacturer’s instructions (Beyotime Institute of Biotechnology), the LDH level in the supernatants was measured at 490 nm with a spectrophotometric microplate reader.

### RNA m6A dot blot assay

Total RNA was extracted using TRIzol reagent from PC9/GR cells transfected with si-NC and si-LINC00969. Within five minutes, the RNA samples were denatured at 65 °C by mixing in three volumes of incubation buffer. Then, the samples (800 ng, 600 ng, 400 ng and 200 ng) were dissolved in SSC buffer (Sigma-Aldrich) and transferred onto a nitrocellulose membrane (Amersham) with a Bio-Dot apparatus (Bio-Rad). After ultraviolet (UV) cross-linking, the membrane was stained with 0.02% methylene blue (Sigma). The membrane was incubated with an anti-m6A antibody (1:2000, Synaptic System, Cat. # 202003) overnight at 4 °C. Dots were visualized by an imaging system (Bio-Rad) after the membrane was incubated for an hour with HRP-conjugated anti-mouse immunoglobulin G (HRP-IgG).

### M6A RNA immunoprecipitation (MeRIP) assay

Total RNA was extracted by TRIzol from PC9/GR cells. MeRIP assays were performed using a Magna MeRIP™ m6A Kit (17-10499, Millipore, MA) following the manufacturer’s recommendations. To enrich m6A-modified mRNA, DNA-free fragmented RNAs were incubated with specific antibody- or rabbit IgG (Millipore)-bound magnetic Dynabeads (Abcam). The locations of specific m6A sites in specific genes were predicted with RMBase v2.0 (http://rna.sysu.edu.cn/rmbase/) and SRAMP (http://www.cuilab.cn/sramp) (forward: GACTTCGTGCAAAGGGCCA; reverse: TGTGGTCCATTCTGGTGGAG). After RNA was extracted from cells, it was subjected to qRT-PCR using specific primers, and RNA levels were compared to the input (10%) RNA levels.

### RNA stability assay

After siRNA transfection of PC9/GR cells for 24 h, 10 µg/ml actinomycin D (Sigma Aldrich, St. Louis, MO, USA) was added to the cells for 0, 4, 8 and 12 h. The cells were harvested and analysed by qRT-PCR.

### Western blot analysis and antibodies

SDS‒PAGE was used to separate proteins in cell lysates, and the proteins were then electrophoretically transferred to PVDF membranes (Millipore), which were then incubated with specific antibodies. Quantitative densitometry was performed on autoradiograms (Quantity One software; Bio-Rad). An anti-GAPDH antibody was used as a control. The anti-EZH2 (dilution ratio, 1:1000, Cat. # ab191250), anti-METTL3 (dilution ratio, 1:1000, Cat. # ab195352) and anti-YTHDF2 (dilution ratio, 1:1000, Cat. # ab220163) antibodies were purchased from Abcam, and the anti-NLRP3 (dilution ratio, 1:1000, Cat. # 15101), anti-caspase 1(dilution ratio, 1:1000, Cat. #3866), anti-cleaved caspase 1(dilution ratio, 1:1000, Cat. # 4199), anti-GSDMD (dilution ratio, 1:1000, Cat. # 96458) and anti-cleaved GSDMD (dilution ratio, 1:1000, Cat. # 36425) antibodies were purchased from Cell Signaling Technology. Each experiment was repeated three times.

### Statistical analysis

SPSS 22.0 software (IBM, SPSS, Chicago, IL, USA) and GraphPad Prism 8.0 software (GraphPad Software, San Diego, CA, USA) were used for all statistical analyses. Student’s *t* test, the X^2^-test, or the Wilcoxon test was used to examine differences between groups. Statistical significance was determined by two-sided *p* value at a probability level of 0.05.

## Supplementary information

Supplemental Material

checklist

Original Data File

## Data Availability

In response to requests from the corresponding author, data supporting these findings can be accessed.
