# Supplementary material for: LncRNA LINC00969 promotes acquired gefitinib resistance by epigenetically suppressing of NLRP3 at transcriptional and posttranscriptional levels to inhibit pyroptosis in lung cancer
Source: Cell Death Dis. 2023 May 8;14(5):312. doi: 10.1038/s41419-023-05840-x (PMC10167249; doi:10.1038/s41419-023-05840-x)
Supplement: Supplementary file 1 — Supplemental Material [file 41419_2023_5840_MOESM1_ESM.pdf]

Supplemental Information

Figure S1

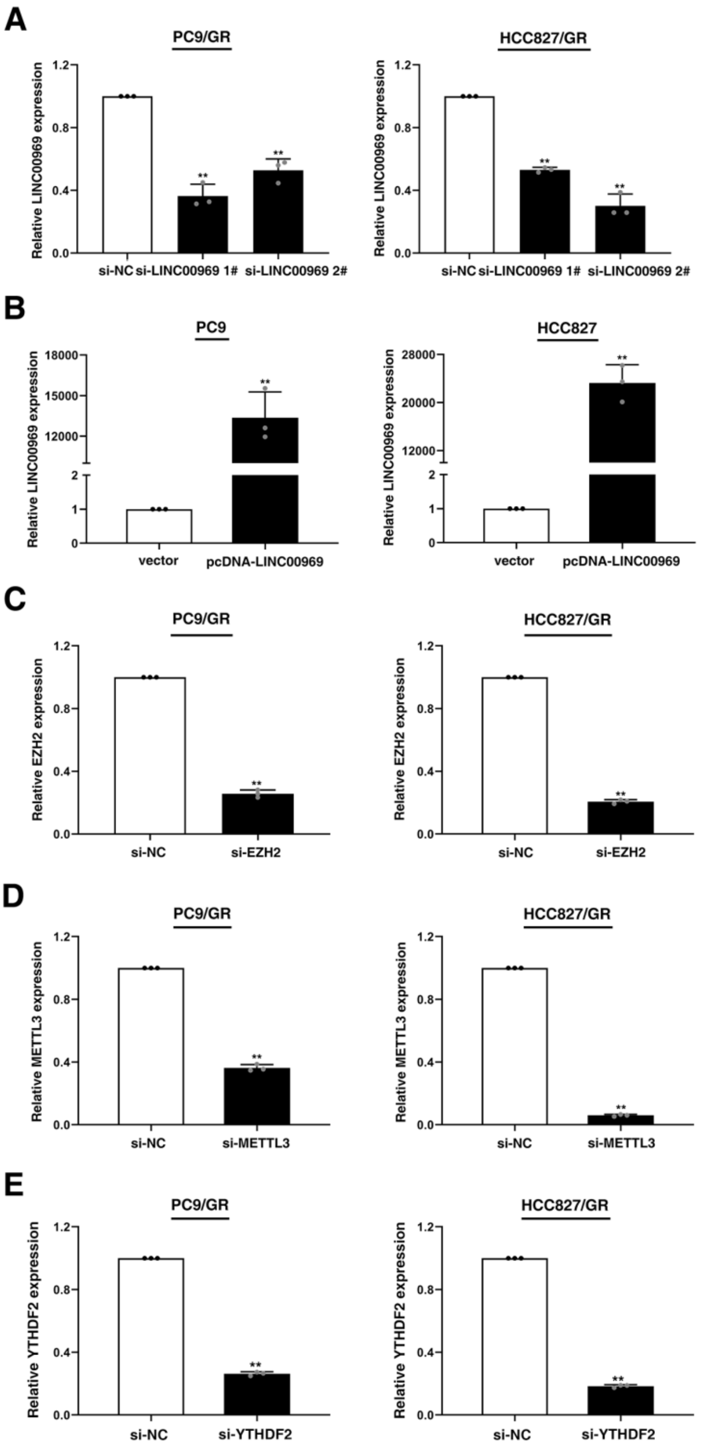

Figure S1. (A) qRT-PCR analysis of LINC00969 expression in PC9/GR and HCC827/GR cells transfected with si-NC or si-LINC00969. (B) qRT-PCR analysis of LINC00969 expression in PC9

and HCC827 cells transfected with overexpression plasmid. (C) qRT-PCR analysis of EZH2 expression in PC9/GR and HCC827/GR cells transfected with the siRNA. (D)The METTL3 expression in PC9/GR and HCC827/GR cells was tested by qRT-PCR. (E) qRT-PCR analysis of YTHDF2 expression in PC9/GR and HCC827/GR cells. **$P < 0.01$.

Table S1: The list of primers and siRNA sequence.

GENE	Forward primer	Reverse primer
**human qRT-PCR**		
**LINC00969**	TGGTGGAAGTGAGACCGAAAT	GTGATCCGTCCCAAGACAGC
**NLRP3**	GATCTTCGCTGCGATCAACAG	CGTGCATTATCTGAACCCCAC
**Caspase1**	GTGCAGGACAACCCAGCTAT	TGCGGCTTGACTTGTCCATT
**GSDMD**	GGGTAGTCCGGAGAGTGGTC	GAGGGCTTCCTAACCACCAG
**EZH2**	TGCACATCCTGACTTCTGTG	AAGGGCATTCACCAACTCC
**METTL3**	CAAGCTGCACTTCAGACGAA	GCTTGGCGTGTGGTCTTT
**YTHDF2**	TAGCCAACTGCGACACATTC	CACGACCTTGACGTTCCTTT
**GAPDH**	GAAGGTGAAGGTCGGAGTC	GAAGATGGTGATGGGATTTC
**ChIP-qPCR primers**		
**LINC00969**	CACCATGTCTGGCCTTGTAGT	AGAATCGAGGACACACTCACC
**NLRP3**	TCTCTCTAGCTTCAGCACCTG	TGAAAGGTGTCATTCGGGGC
**MeRIP-qPCR primers**		
**NLRP3**	GACTTCGTGCAAAGGGCCA	TGTGGTCCATTCTGGTGGAG
**Sequences for siRNAs**		
**si-LINC00969 1#**	AAGUGAGACCGAAAUAGACUCUGAA	
**si-LINC00969 2#**	GAGCAUGGUUCAGUGAGUUCUCCAA	
**si-EZH2**	GAGGUUCAGACGAGCUGAUUU	
**si-METTL3**	CTGCAAGTATGTTCACTATGA	
**Si-YTHDF2**	AGGACGTTCCCAATAGCCAA	
