# Supplementary material for: LncRNA LINC00969 promotes acquired gefitinib resistance by epigenetically suppressing of NLRP3 at transcriptional and posttranscriptional levels to inhibit pyroptosis in lung cancer
Source: Cell Death Dis. 2023 May 8;14(5):312. doi: 10.1038/s41419-023-05840-x (PMC10167249; doi:10.1038/s41419-023-05840-x)

Figure 3B PC9 PC9/GR

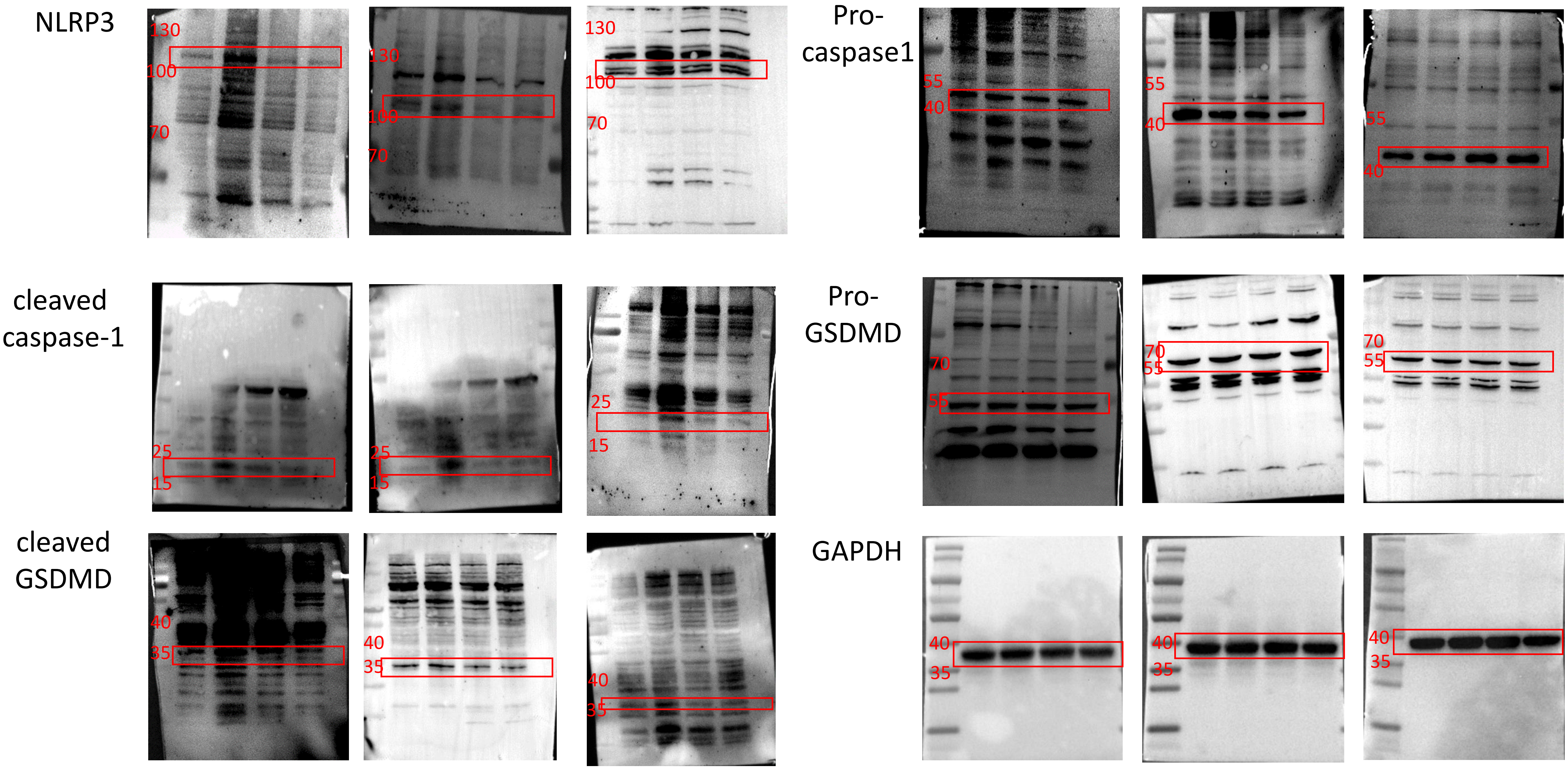

Figure 3B HCC827 HCC827/GR

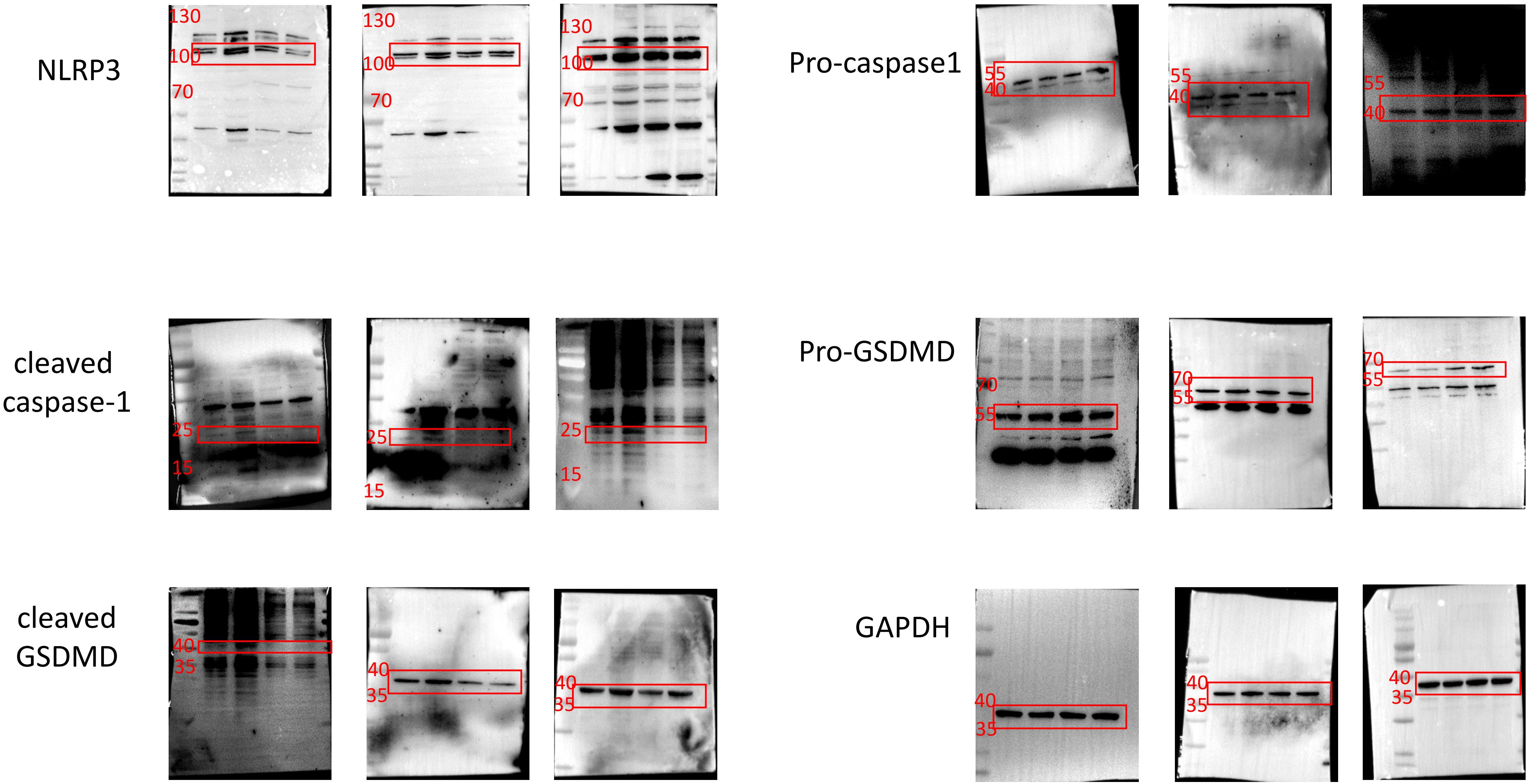

Figure 3D PC9/GR

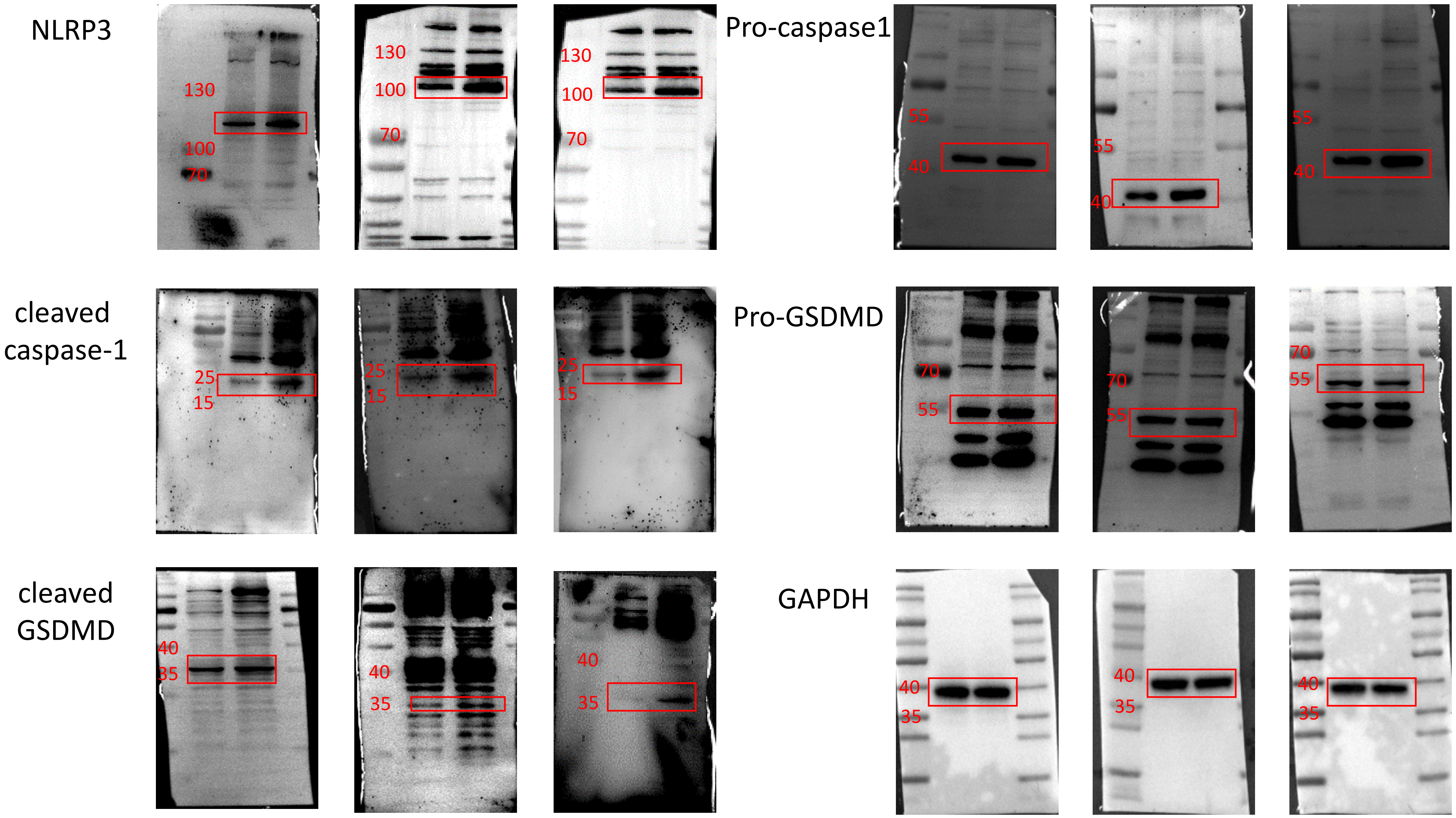

Figure 3D HCC827/GR

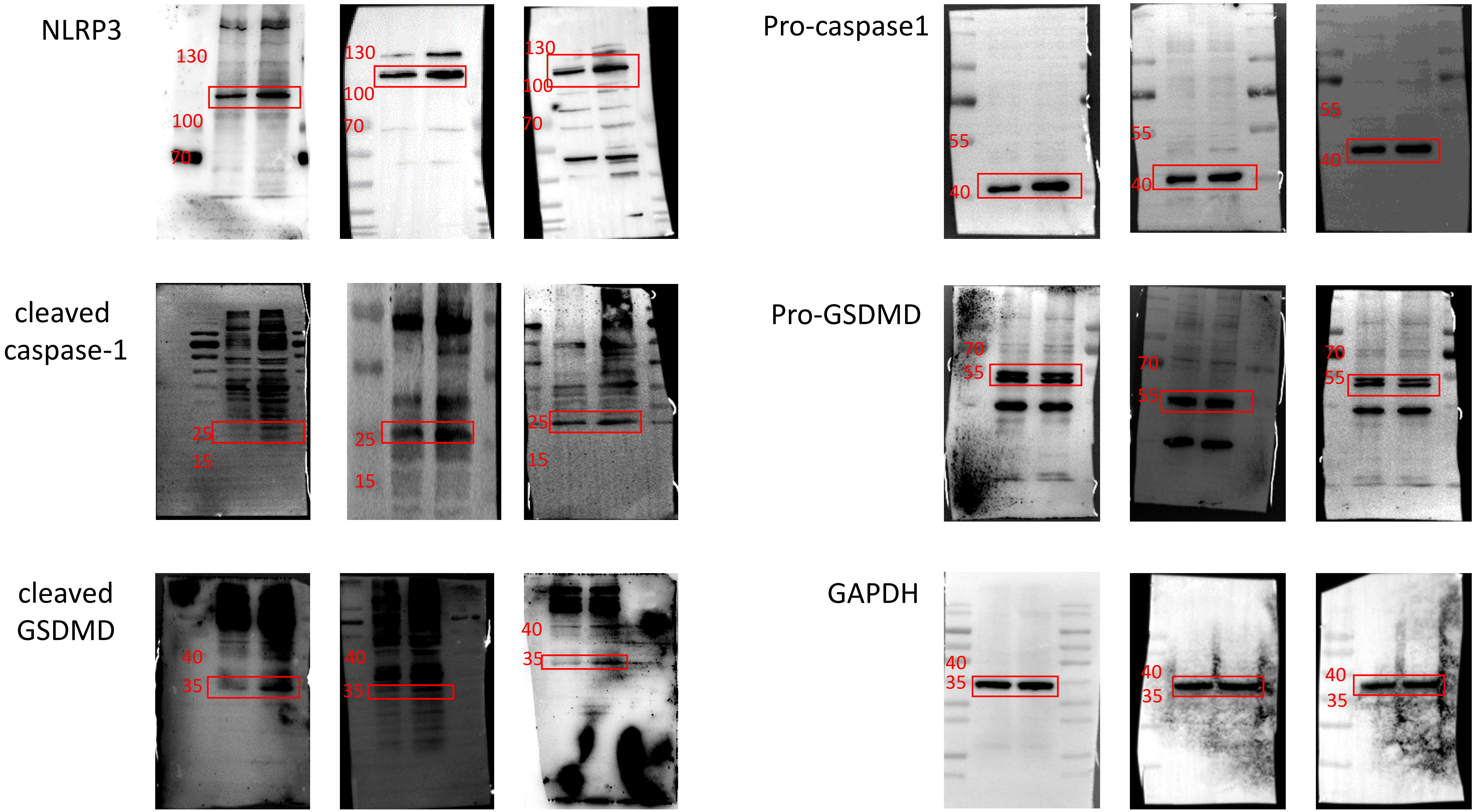

Figure 4E

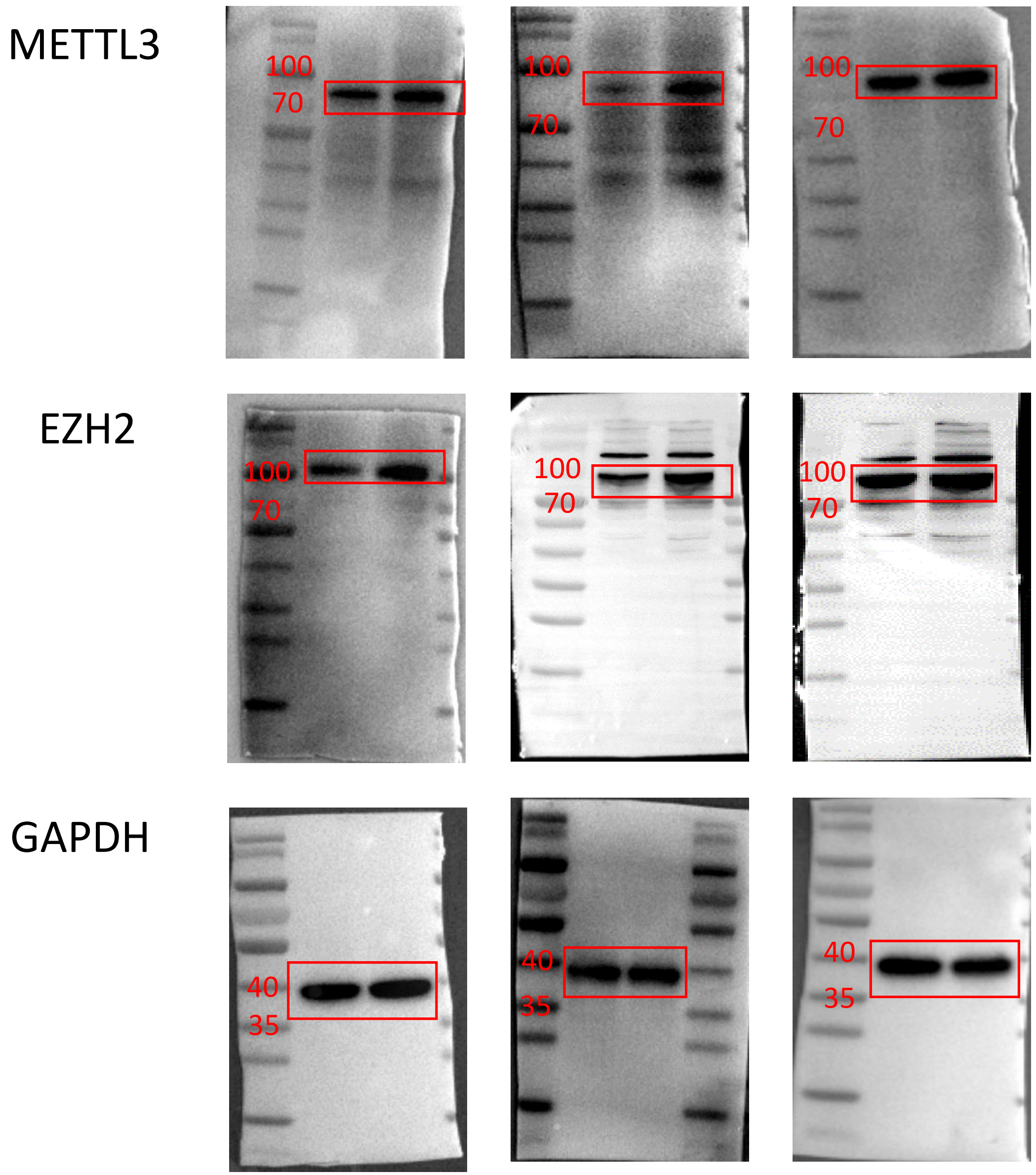

Figure 4M

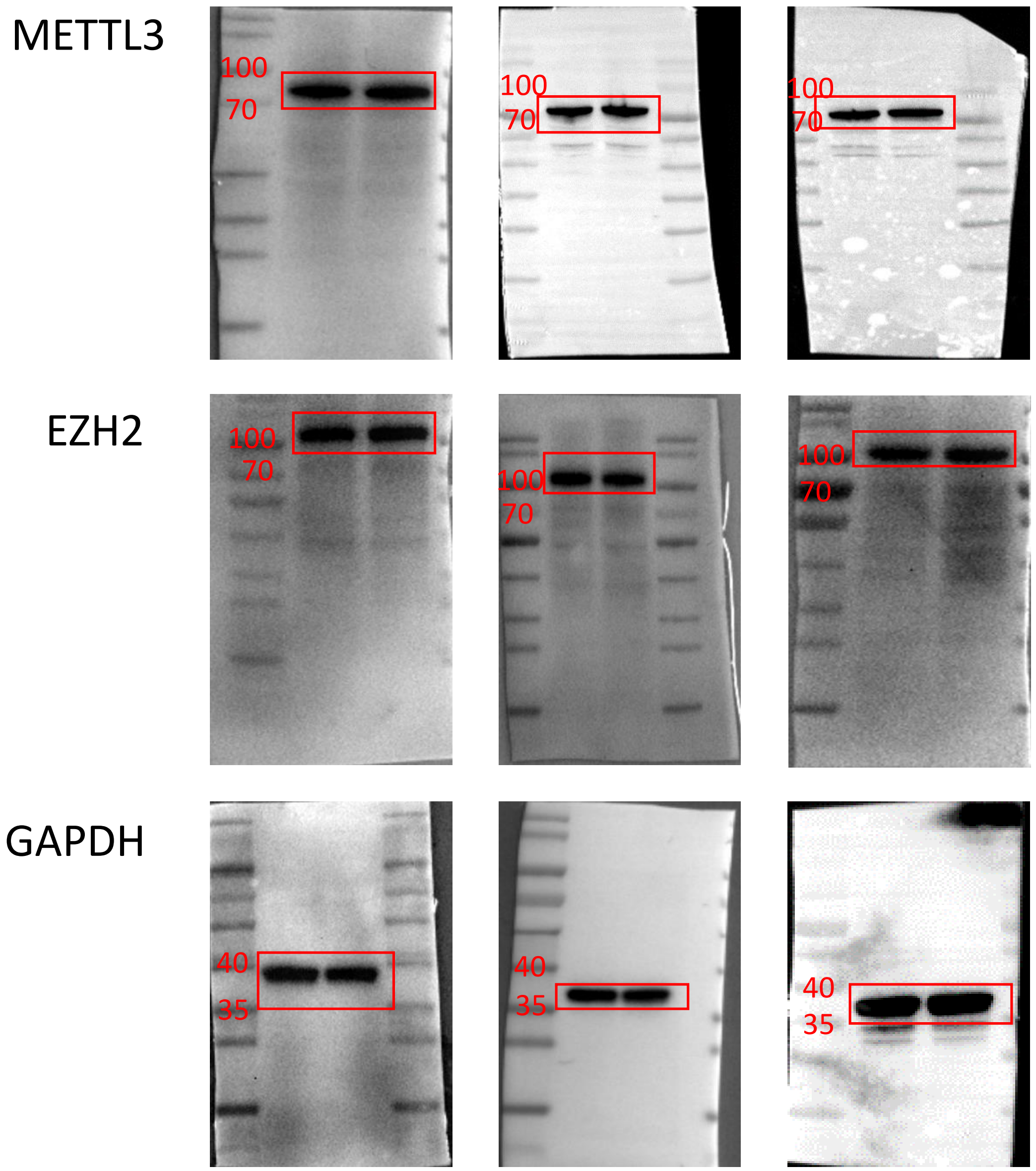

Figure 5B PC9/GR

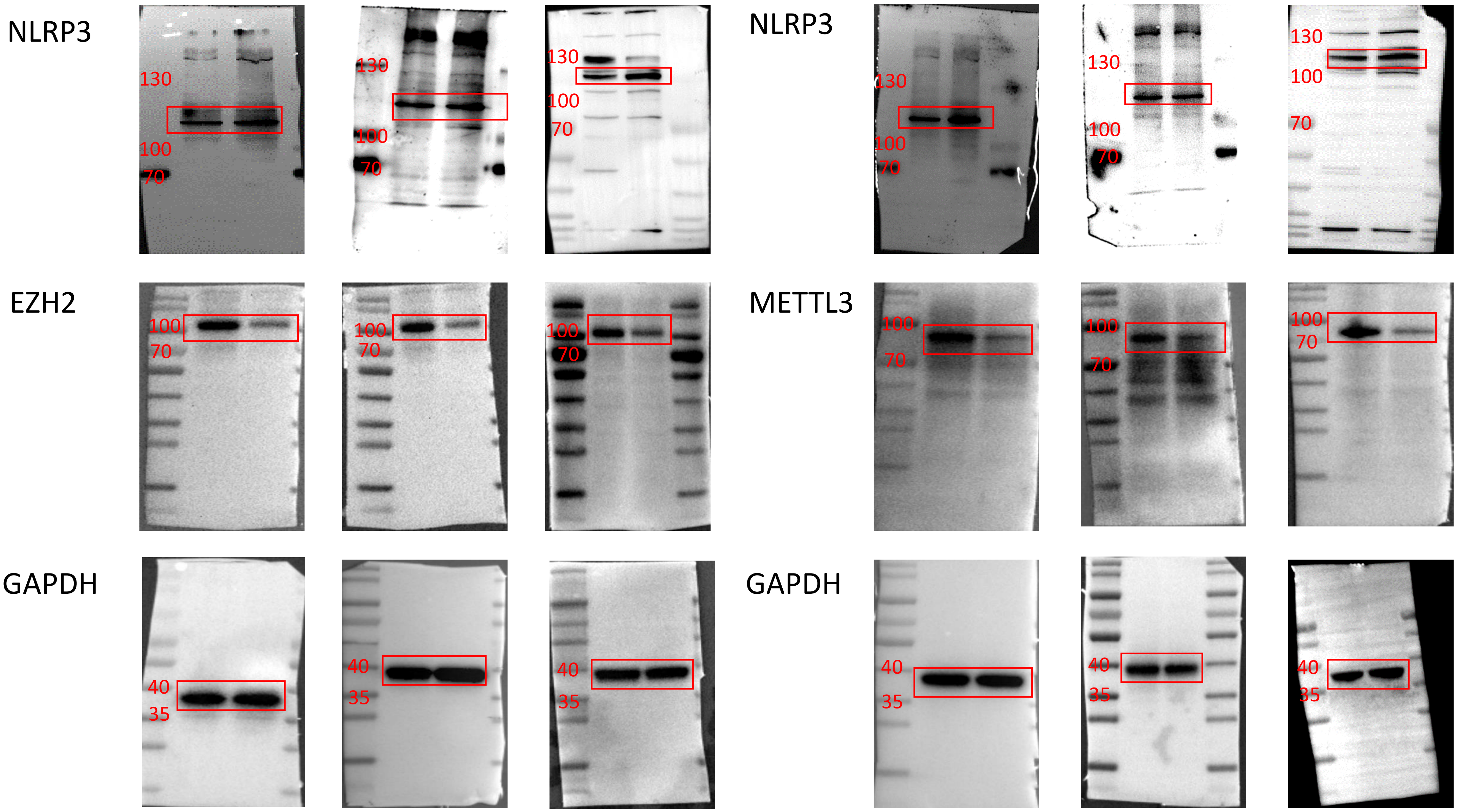

Figure 5B HCC827/GR

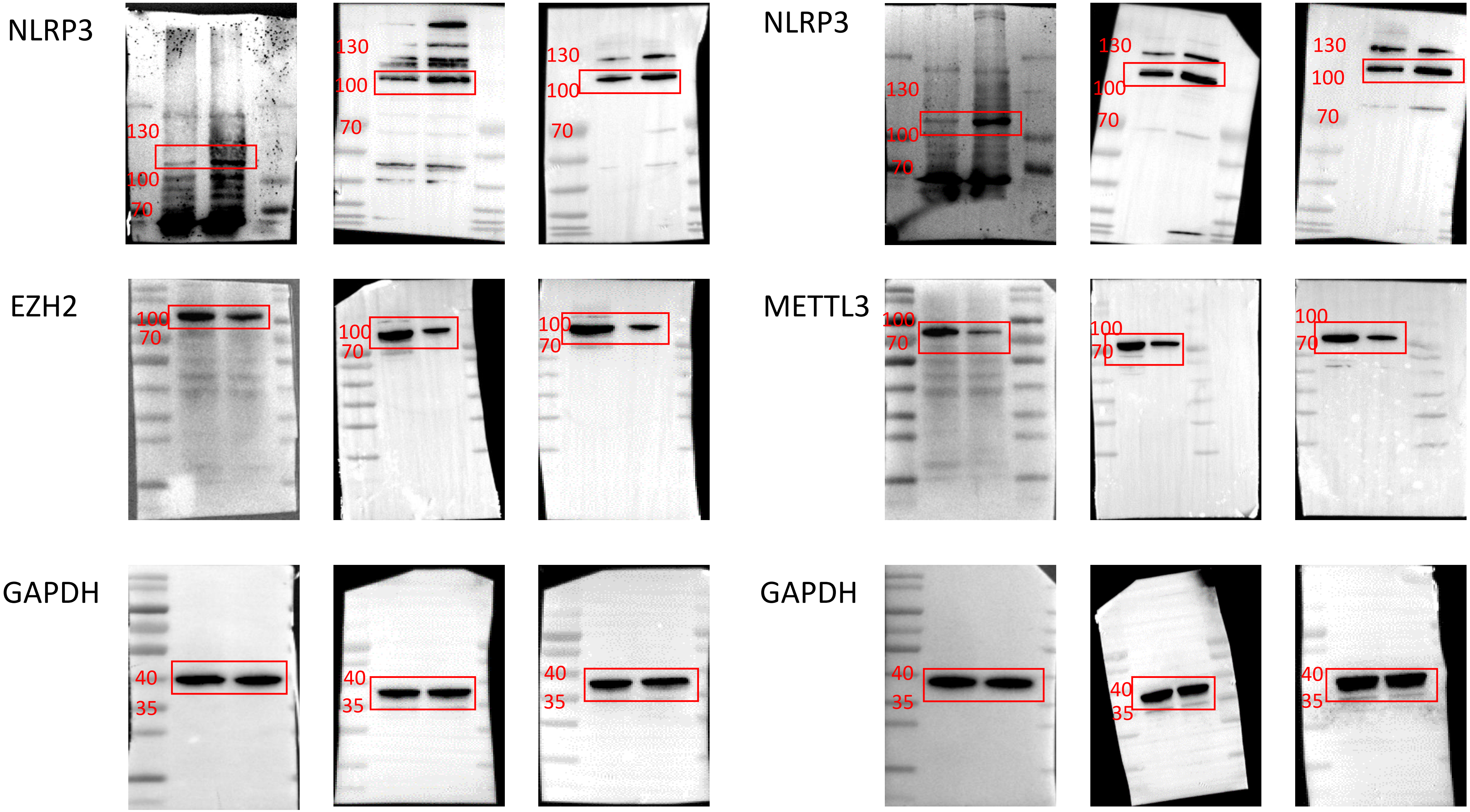

Figure 5D PC9/GR

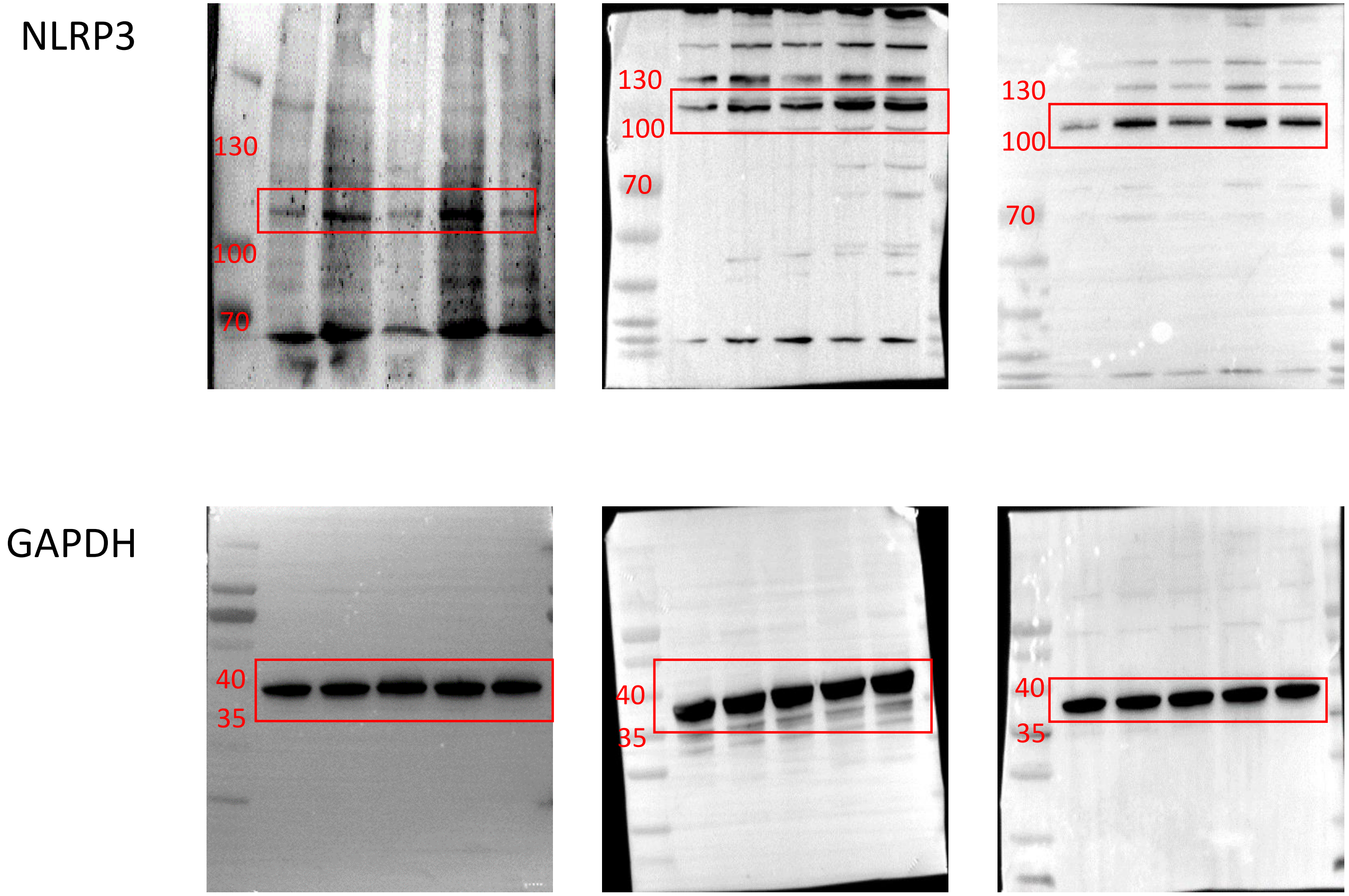

Figure 5D HCC827/GR

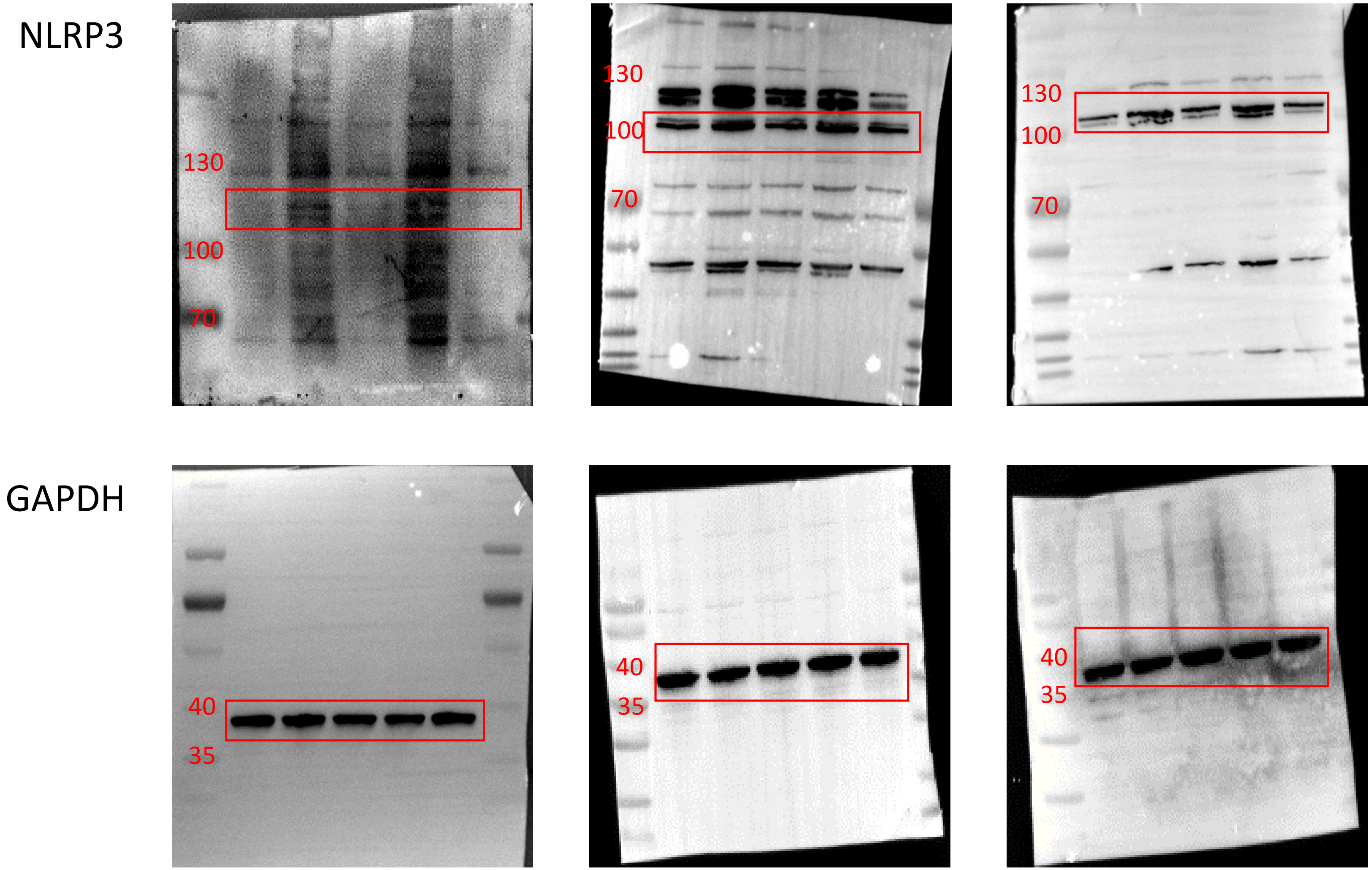

Figure 6B PC9 PC9/GR

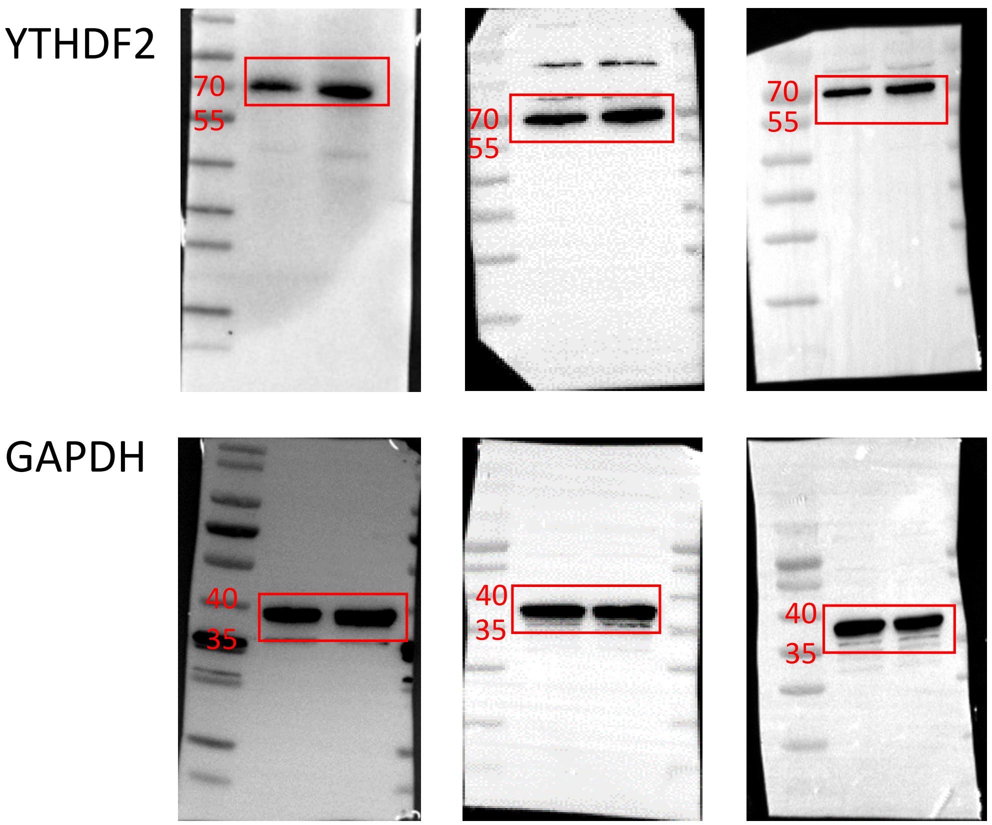

Figure 6F PC9/GR

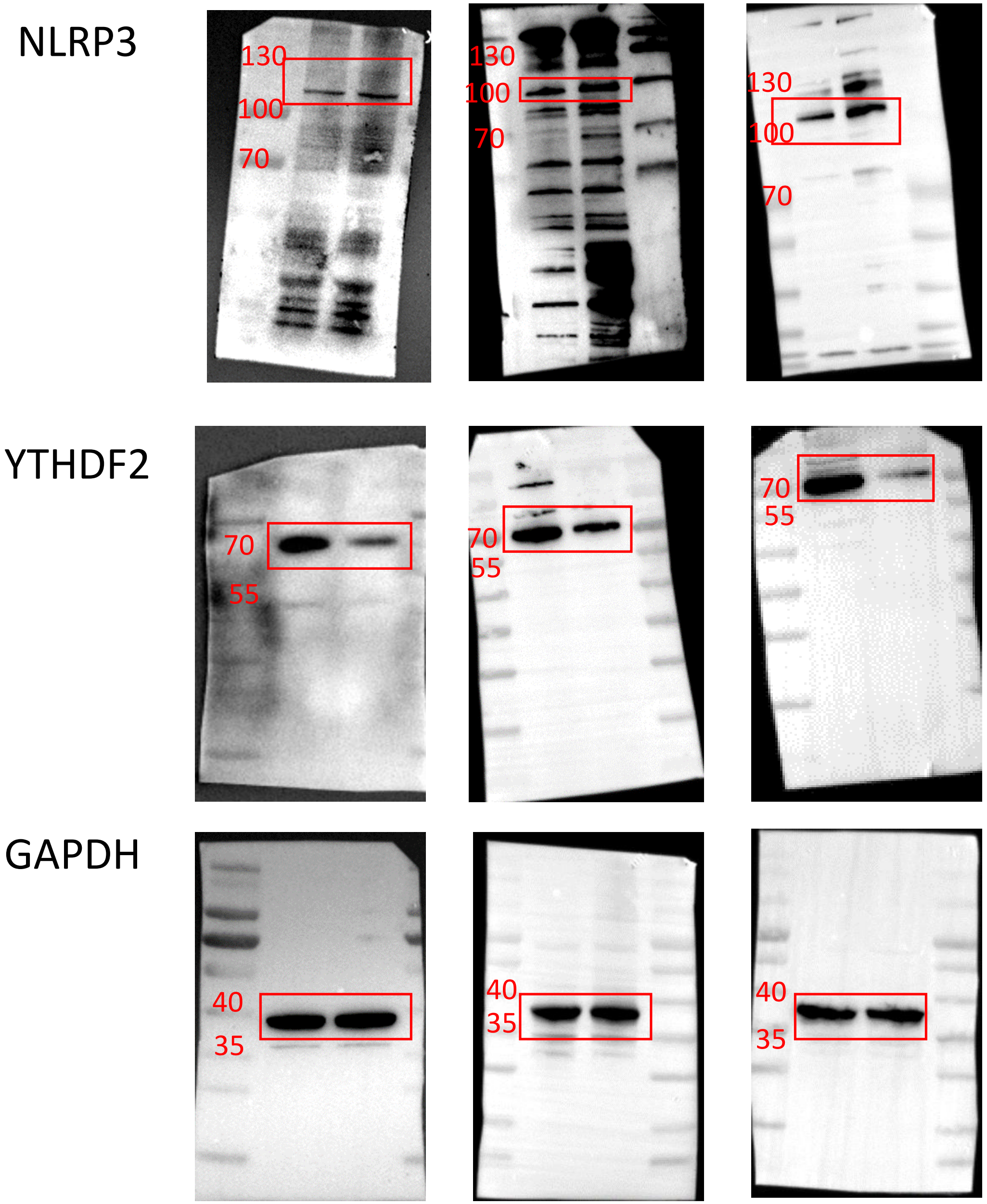

Figure 6B HCC827 HCC827/GR

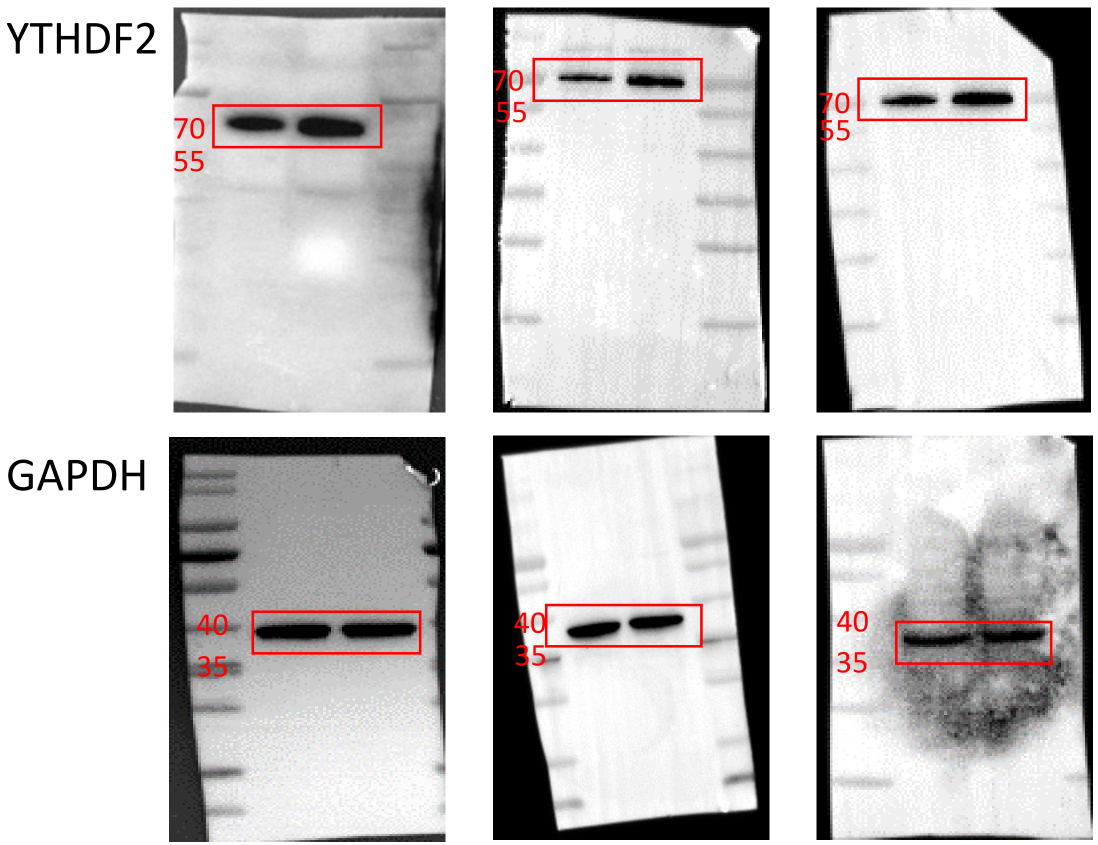

Figure 6F HCC827/GR

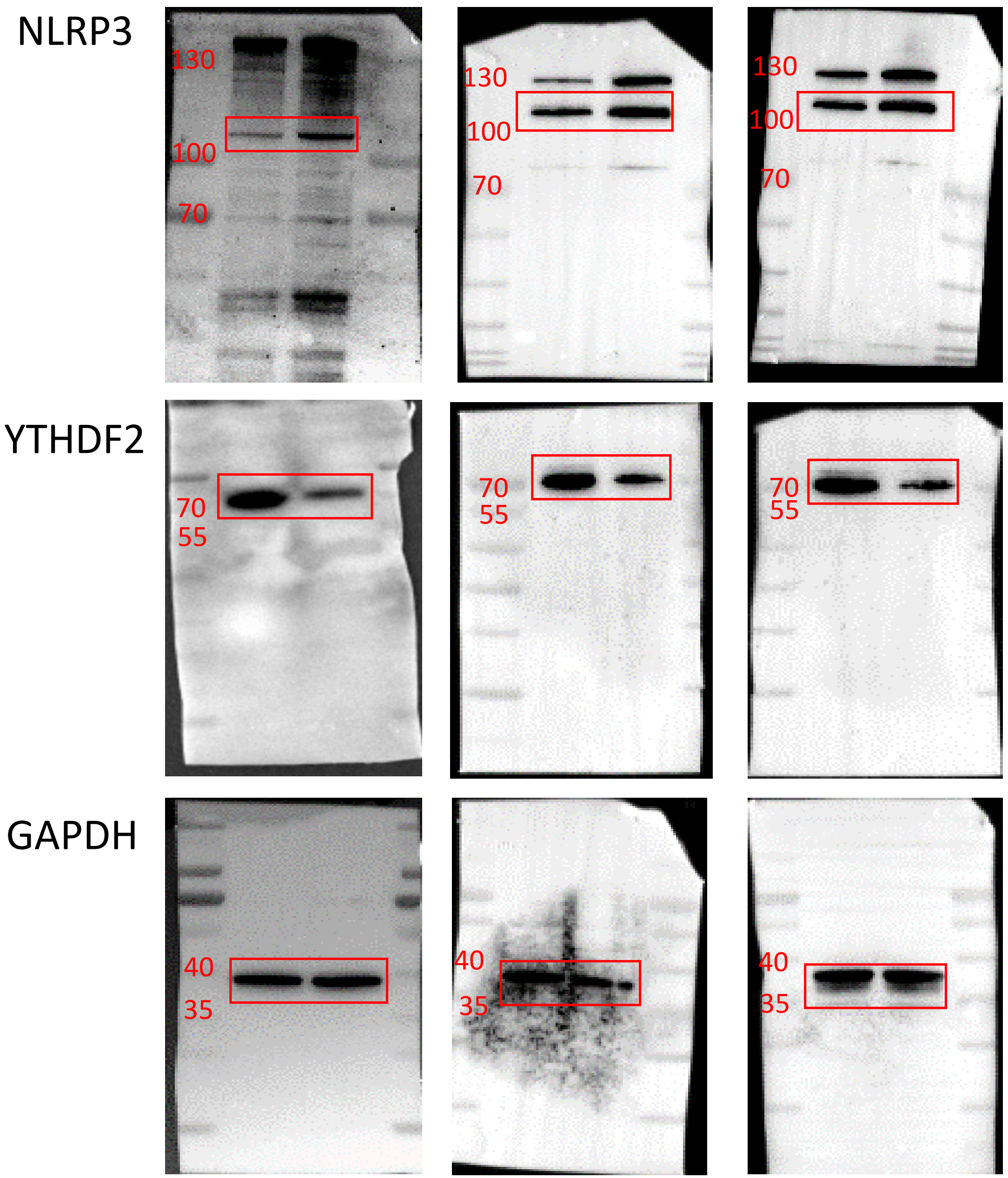

Supplement: Supplementary file 3 — Original Data File [file 41419_2023_5840_MOESM3_ESM.pdf]
